# Global decline in microbial-derived carbon stocks with climate warming and its future projections

**DOI:** 10.1093/nsr/nwae330

**Published:** 2024-09-16

**Authors:** Yuting Liang, Han Hu, Thomas W Crowther, Rainer Georg Jörgensen, Chao Liang, Ji Chen, Yishen Sun, Chaoyang Liu, Jixian Ding, Aidi Huang, Jizhong Zhou, Jiabao Zhang

**Affiliations:** State Key Laboratory of Soil and Sustainable Agriculture, Institute of Soil Science Chinese Academy of Sciences, Nanjing 210008, China; University of the Chinese Academy of Sciences, Beijing 100049, China; State Key Laboratory of Soil and Sustainable Agriculture, Institute of Soil Science Chinese Academy of Sciences, Nanjing 210008, China; University of the Chinese Academy of Sciences, Beijing 100049, China; Department of Environmental Systems Science, ETH Zurich, Zurich 8092, Switzerland; Department of Soil Biology and Plant Nutrition, University of Kassel, Kassel 34117, Germany; Institute of Applied Ecology, Chinese Academy of Sciences, Shenyang 110016, China; Key Lab of Conservation Tillage and Ecological Agriculture, Shenyang 110016, China; Department of Agroecology, Aarhus University, Tjele 8830, Denmark; Aarhus University Centre for Circular Bioeconomy, Aarhus University, Tjele 8830, Denmark; iCLIMATE Interdisciplinary Centre for Climate Change, Aarhus University, Roskilde 4000, Denmark; State Key Laboratory of Soil and Sustainable Agriculture, Institute of Soil Science Chinese Academy of Sciences, Nanjing 210008, China; University of the Chinese Academy of Sciences, Beijing 100049, China; State Key Laboratory of Soil and Sustainable Agriculture, Institute of Soil Science Chinese Academy of Sciences, Nanjing 210008, China; State Key Laboratory of Soil and Sustainable Agriculture, Institute of Soil Science Chinese Academy of Sciences, Nanjing 210008, China; State Key Laboratory of Soil and Sustainable Agriculture, Institute of Soil Science Chinese Academy of Sciences, Nanjing 210008, China; University of the Chinese Academy of Sciences, Beijing 100049, China; School of Biological Sciences, University of Oklahoma, Norman, OK73019, USA; State Key Laboratory of Soil and Sustainable Agriculture, Institute of Soil Science Chinese Academy of Sciences, Nanjing 210008, China

**Keywords:** stable soil organic carbon, microbial-derived carbon, climate change, terrestrial carbon cycle

## Abstract

Soil organic carbon (SOC) represents the largest terrestrial pool of organic carbon and is indispensable for mitigating climate change and sustaining soil fertility. As a major component of stable SOC, microbial-derived carbon (MDC) accounts for approximately half of the total SOC and has repercussions on climate feedback. However, our understanding of the spatial and temporal dynamics of MDC stocks is limited, hindering assessments of the long-term impacts of global warming on persistent SOC sequestration in the soil‒atmosphere carbon cycle. Here, we compiled an extensive global dataset and employed ensemble machine learning techniques to forecast the spatial-temporal dynamics of MDC stocks across 93.4% of the total global land area from 1981 to 2018. Our findings revealed that for every 1°C increase in temperature, there was a global decrease of 6.7 Pg in the soil MDC stock within the predictable areas, equivalent to 1.4% of the total MDC stock or 0.9% of the atmospheric C pool. Tropical regions experienced the most substantial declines in MDC stocks. We further projected future MDC stocks for the next century based on shared socioeconomic pathways, showing a global decline in MDC stocks with a potential 6–37 Pg reduction by 2100 depending on future pathways. We recommend integrating the response of MDC stocks to warming into socioeconomic models to enhance confidence in selecting sustainable pathways.

## INTRODUCTION

Soil organic carbon (SOC) constitutes the largest reservoir of organic carbon in terrestrial ecosystems [1], surpassing the combined total found in vegetation and the atmosphere [2,3]. The role of SOC is multifaceted, just like its name, Storing carbon, Offering food, and Conserving ecosystem health [3,4]. Current understanding suggests that plant-derived, readily degradable carbon compounds undergo a sequence of microbially mediated processes within the soil, including energy-yielding mineralization (catabolism) and incorporation into microbial biomass (anabolism) [4,5]. The subsequent death and lysis of microbial cells, along with the release of non-biomass metabolites such as extracellular enzymes, extracellular polymeric substances, signaling molecules, and antibiotics [6], results in the production of various organic cellular compounds, including cell envelope fragments and small biopolymers. These compounds can resist reutilization by subsequent generations of microbes, bind to minerals, and contribute to the formation of the slowly cycling pool of SOC [3,4]. This fraction, known as microbial-derived carbon (MDC), originates from microbial necromass [4,7]. Compared with plant-derived carbon, MDC is characterized by a more recalcitrant chemical structure [8,9] and has a greater affinity for minerals and metal oxides [10,11], making it a crucial component of the long-term stable SOC pool. It accounts for approximately half of the total SOC [4,11] and represents a dynamic ecosystem component with repercussions on climate feedback. Comprehensive evaluations of MDC stock dynamics over the long term are essential for predicting soil carbon storage capacity under future climate scenarios. Such assessments are vital for ensuring and supporting the continued functioning of terrestrial ecosystems.

The analysis of amino sugars has become the preeminent method for estimating microbial-derived carbon stocks in soils [4,11–13], offering a potential pathway to scale up standardized *in situ* measurements for continental and global assessments [11,13,14]. Amino sugars serve as essential biomarkers of microbial cell walls that accumulate in soil following cell lysis [7,11]. Soil scientists have extensively studied a diverse range of natural and artificial ecosystems worldwide, providing comprehensive spatial data coverage [15]. This database is instrumental in predicting the spatial distribution of MDC stocks. However, longitudinal data on changes in MDC stocks over time are scarce. Furthermore, MDC accumulation is subject to the influence of geo-biochemical processes and specific environmental conditions [14,16–18], which complicate the generalization of observed spatial patterns and the extension of these patterns into a spatiotemporal model of MDC dynamics. Fortunately, advances in machine learning techniques offer a promising solution to this challenge. With sufficient observations from varying temporal points and global environmental coverage, it is feasible to model the spatiotemporal dynamics of MDC stocks and make informed extrapolations at a global scale [19]. Once such a model is established, we can identify regions likely to experience changes and develop targeted management and conservation strategies for climate-sensitive areas.

To construct the spatial-temporal model of MDC stocks, we conducted a comprehensive data collection effort, including data on amino sugars, soil nutrients, and microbial community structure over a 25-year period. Utilizing ensemble machine learning approaches, we leveraged the dataset in conjunction with global environmental coverage data to generate predictions of MDC stocks spanning from 1981 to 2018. We hypothesize that increases in global temperature due to climate warming are leading to a significant decline in MDC stocks with tropical regions experiencing the most substantial reductions. This trend is expected to continue into the future, depending on different socioeconomic pathways. Such information is essential if we are to generate a general basis for modeling and forecasting the coupled atmosphere‒soil carbon cycle in a warming climate.

## PERFORMANCE OF MDC STOCKS IN RESPONSE TO GLOBAL WARMING: A HISTORICAL ANALYSIS

**Figure 1.**
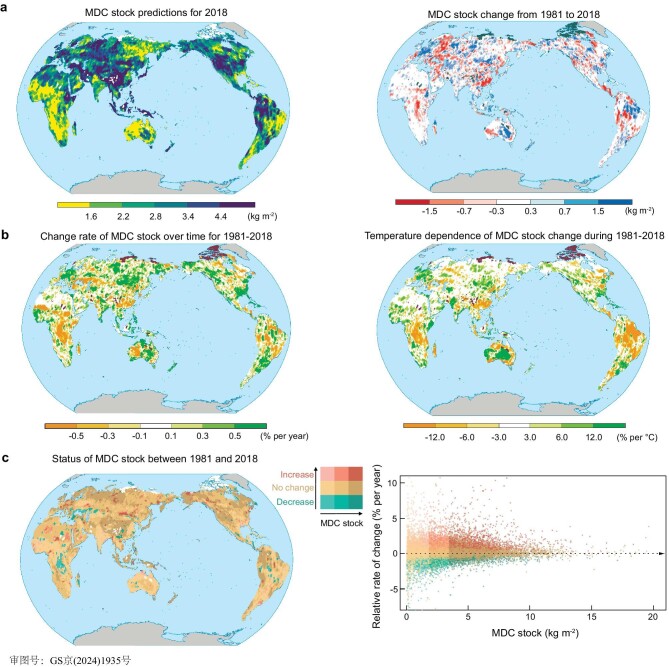
Predicted spatial distributions and temporal trends of microbial-derived carbon (MDC) stocks. (a) Global map of the predicted MDC stock distribution for the year 2018, along with the changes from 1981 to 2018. The white areas in the left panel and the dark areas in the right panel are the areas with low confidence projections. (b) The relative rates of change in MDC stocks as percentages per annum and per degree Celsius. The dark areas are the areas with low confidence projections. (c) Status of MDC stocks between 1981 and 2018. Bivariate plot comparing the relative rate of change in MDC stock (% per year) against the quantity of MDC stocks. The status categories for the rate of change were determined using confidence intervals, while the MDC stock status groups were established based on quantile distributions (divided into three equal parts). The white areas are the areas with low confidence projections.

Using an array of databases and ensemble machine learning approaches (Figs S1–S10), we produced annual predictions of MDC stocks globally within the topsoil layer (0–30 cm) spanning from 1981 to 2018 (Fig. 1; details in Supplementary Data). These predictions take into account the temporal and spatial variations in climate, soil properties, vegetation, and microbial communities. The representative analyses revealed that our model was capable of predicting the MDC stocks with medium-to-high confidence across 93.4% of the total global land area (Fig. S11). Low-confidence prediction areas are primarily located in the Arctic Circle and the Tibetan Plateau.

**Figure 2.**
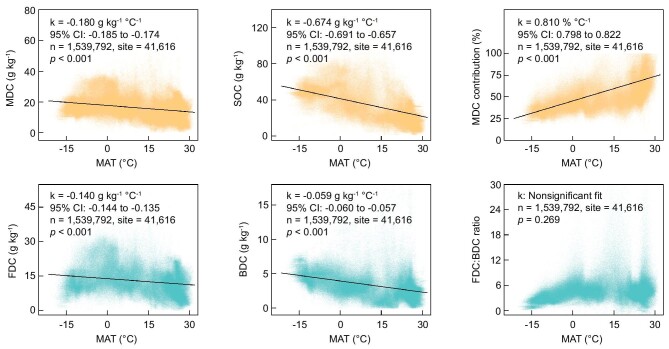
The impact of warming on microbial-derived carbon (MDC) concentrations. The analysis was conducted using linear mixed-effects (LME) models, where temperature change served as a fixed effect and site served as a random effect. The slopes of the solid lines fitted to the data represent the magnitude of the warming impact, measured in grams per kilogram per degree Celsius (g kg^−1^°C^−1^). The variable *k* denotes the slope parameter of the LME models, while the 95% confidence interval (CI) indicates that the range within the true slope is likely to lie based on the model output. The term *n* signifies the total number of observations included in the analysis, and ‘site’ refers to the number of distinct locations sampled. MAT, mean annual air temperature; BDC, bacterial-derived carbon; FDC, fungal-derived carbon; SOC, soil organic carbon.

To elucidate the impact of global warming on MDC stocks, we employed a linear mixed-effects (LME) model incorporating mean annual temperature (MAT) as a fixed effect and site as a random effect on the slope and intercept (Table S1). The rationale for treating site as a random effect is to ensure that changes in MAT are driven by time rather than confounded by geographical differences. Our LME model findings indicate that a 1°C increase in MAT correlates with a global average decrease in the MDC concentration of 0.180 g kg^−1^ (95% CI: 0.174 to 0.185; Fig. 2). This translates to an estimated decrease of 6.7 Pg in global topsoil MDC stocks per 1°C increase in MAT, which equates to ∼1.4% of the total global MDC stock or 0.9% of the atmospheric carbon pool [1]. Notably, our model also revealed that a 1°C increase in MAT correlated with a global average decrease in SOC concentration of 0.674 g kg^−1^ (95% CI: 0.657 to 0.691; Fig. 2). This implies that under global warming conditions, the change in MDC contributes only 27% to the variation in SOC, despite MDC stocks accounting for more than half of SOC stocks on a global scale.

Furthermore, there is considerable biochemical and biophysical diversity in soil carbon derived from various microbial sources. Several studies have suggested that fungal necromass is more resistant to decomposition than bacterial necromass because of its higher C:N stoichiometry [4,9], the presence of complex interlinked polysaccharides in fungal cell walls, and the formation of tannins [11,20,21]. If this premise holds true, the ratio of fungal-derived carbon (FDC) to bacterial-derived carbon (BDC) could serve as an indicator for assessing the biological resilience of MDC against decomposition. Our results revealed that with increasing temperature, the rate of change in FDC concentrations exceeded that of BDC concentrations, as indicated by nonoverlapping 95% confidence intervals (Fig. 2). However, given that the global FDC stocks are approximately four times larger than the BDC stock, the global average FDC:BDC ratio does not significantly change with temperature (*P* = 0.269, Fig. 2). Collectively, our findings suggest that global warming significantly diminishes MDC stocks without substantially affecting their biological resistance on a global scale.

To elucidate the spatially explicit temperature dependencies of MDC stocks, we computed the relative percentage change for each site over the period from 1981 to 2018 (Fig. 1b). Our findings indicate that regions with higher MDC stocks generally exhibited lower rates of relative change (Fig. 1c). Notably, tropical climates experienced the most substantial MDC stocks depletion, with an average loss rate of 0.199 kg m^−2^ (95% CI: 0.193 to 0.205) or 7.3% of the total regional stocks per degree Celsius of warming (Table S2). The Amazon River basin, southeastern Asia, and central Africa were among the areas with the highest rates of MDC loss (Fig. 1b). In contrast, in cold and polar climates, MDC stocks significantly increased in response to warming. The respective average rates of MDC increase were 0.004 kg m^−2^ (95% CI: 0.004 to 0.005) and 0.001 kg m^−2^ (95% CI: 0.000 to 0.002) for cold and polar climates per degree Celsius of warming, which equates to 0.1% of the total MDC stocks in these climates (Table S2). This increase was primarily attributed to an increase in FDC stocks, as FDC concentrations significantly increased (*P* < 0.001; Table S3), while BDC concentrations significantly decreased (*P* < 0.001; Table S4) in these climates. Consequently, the FDC:BDC ratio increased (*P* < 0.001; Table S5), suggesting that warming enhanced the biological resistance of MDC in these regions. A possible explanation is that fungi are more competitive than bacteria in the long term in assimilating warming-induced fresh plant-derived C inputs [22]. As a result, MurA is more likely to be degraded to compensate for microbial demand, while GlcN tends to accumulate in MDC stocks [22,23].

The overall trend thus revealed a ‘Matthew effect’ on MDC stocks with increasing temperature: tropical regions, which are already low in stock, experienced notable reductions, whereas cold and polar regions, which are initially high in stock, experienced simultaneous increases in both stock quantity and biological resistance (Table S2). Beyond these general observations, predictions varied at the local scale, influenced by factors including soil properties, vegetation, climate, and microbial communities, which differed even between neighboring sites and impacted the observed trends [19]. This variability was particularly pronounced in North America, South America, and Oceania, where adjoining sites displayed both increases and decreases in MDC stocks (Fig. 1b).

## THE INFLUENCE OF SOCIOECONOMIC PATHWAY ON MDC STOCKS: A FUTURE OPTION

**Figure 3.**
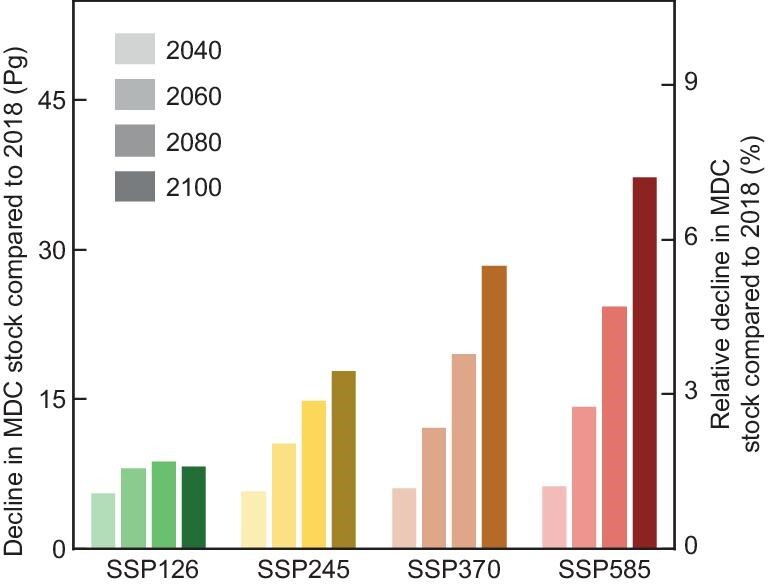
Projections of the total global microbial-derived carbon (MDC) stock under various shared socioeconomic pathways. SSP126 represents a sustainability scenario in which the world is gradually transitioning toward sustainable practices. SSP245, the middle-of-the-road scenario, maintains trends closely aligned with historical patterns. SSP370, the regional rivalry scenario, emphasizes domestic or regional issues due to increased nationalism and conflicts. Finally, SSP585 is a fossil-fueled development scenario characterized by large-scale exploitation of fossil fuels that intensifies environmental degradation and climate change.

To forecast the global distribution of MDC stocks in the future, we employed the Coupled Model Intercomparison Project (Phase 6) and the Shared Socioeconomic Pathway (SSP) to anticipate forthcoming patterns in global MAT [24,25]. Our approach was predicated on the hypothesis that the temperature dependency of MDC stocks would remain consistent throughout the coming century, as observed over recent decades. With this premise, we simulated future global MDC stocks distributions under various SSP scenarios: the sustainability pathway (SSP126), the middle-of-the-road scenario (SSP245), the regional rivalry scenario (SSP370), and the fossil-fueled development scenario (SSP585). Our findings revealed that the choice of socioeconomic pathway substantially shapes the future of MDC stocks (Fig. 3), particularly in tropical regions (Fig. S12). Under the sustainability scenario, a reversal of the declining trend in global MDC stocks is anticipated, with an upward shift expected by 2080–2100. Conversely, under the fossil-fueled development scenario, a decrease of 8% in global MDC stocks from current levels by the year 2100 is projected, with potential reductions of up to 46% in tropical climates (Fig. S12).

Decreases in MDC stocks are likely to precipitate a cascade of ecological repercussions. Primarily, MDC is critical for the long-term sequestration and stabilization of SOC [3,4,11]. A decrease in MDC implies that a significant fraction of stable SOC could be released into the atmosphere as CO_2_, fostering a positive feedback loop between atmospheric and soil carbon pools. In contrast to plant-derived carbon compounds such as lignin and phenols, microbial-derived molecules such as hexoses and amino sugars have extended mean residence times in soils [11,26]. This suggests that once depleted, replenishing MDC stocks will require a prolonged period, potentially spanning several decades or more [4,11], indicating that the depletion of MDC may have lasting effects on the carbon cycle. Second, MDC plays a vital role in sustaining soil fertility and agroecological sustainability [27]. Soil organic matter is fundamental for retaining moisture and nutrients [28], and it is closely correlated with crop biomass production [29]. The degradation of MDC may lead to premature nutrient release [30] and diminished availability of micronutrients [31], both of which are crucial for plant growth. Thus, a reduction in MDC stocks in cultivated lands could increase the risk of decreased crop yields. Third, specific MDC compounds are essential for maintaining soil health and ecological services. For instance, glucosamine and galactosamine can modulate soil pH, and extracellular polymeric substances [32,33] and certain microbial-derived carbohydrates [11] can bind with iron (hydr)oxides to form soil aggregates. A reduction in these MDC compounds leads to decreased soil aggregate stability, reduced pH buffering capacity, and diminished retention of water and nutrients, potentially resulting in soil erosion, limited plant growth, and reduced microbial diversity. Therefore, we advocate for the pursuit of sustainable practices aimed at maintaining MDC stocks to mitigate these ecological outcomes.

In summary, our results offer an empirical foundation for refining the temperature dependency of MDC stocks within atmosphere‒soil carbon cycle models. By extrapolating trends from past decades, it is anticipated that global warming will lead to a decrease in global MDC stocks, which could have severe ecological repercussions for climate change, food security, and ecosystem integrity. To mitigate these impacts, it is critical to implement judicious policies and oversight aimed at fostering the accumulation and preservation of MDC. Such initiatives should encompass a transition to sustainable energy sources [25], prudent land use and agricultural practices [27,34], the establishment of baseline greenhouse gas emissions, and efforts to reduce deforestation [35] and wetland degradation [36]. We advocate for the integration of MDC stocks responses to warming into existing socioeconomic models to bolster confidence in selecting sustainability pathways. Furthermore, additional research is necessary to elucidate the underlying mechanisms driving MDC dynamics under different climate scenarios and management approaches, thereby developing effective strategies to alleviate the impacts of climate change on soil health and ecosystem services.

## METHODS

Detailed descriptions of the methods are available in the online Supplementary Data.

## Supplementary Material

nwae330_Supplemental_Files

## Data Availability

The code for this study is available in the online Supplementary Data.
