# Supplementary material for: Global decline in microbial-derived carbon stocks with climate warming and its future projections
Source: Natl Sci Rev. 2024 Sep 16;11(11):nwae330. doi: 10.1093/nsr/nwae330 (PMC11495487; doi:10.1093/nsr/nwae330)
Supplement: nwae330_Supplemental_Files [file nwae330_supplemental_files.zip › Supplementary_materials.docx]

Supplementary Materials for

**Global decline in stable soil organic carbon stocks with climate warming and its future projections**

Yuting Liang *et al.*

**This file includes:**

Materials and Methods

References for Materials and Methods

Supplementary Figs. 1 to 13

Supplementary Tables 1 to 6

**Other Supplementary Materials not included in this file includes:**

1_Data availability (data file, xlsx)

2_Code for LME model (R code, txt)

3_Code for machine learning (python code, zip)

**Materials and Methods**

**Description of the datasets.** Using the Web of Science (http://apps.webofknowledge.com), Google Scholar (https://scholar.google.com), and the China National Knowledge Infrastructure Database (http://www.cnki.net), we searched for peer-reviewed articles published before October 1, 2023. This process resulted in the collection of five datasets.

The first dataset is composed of the muramic acid (MurA) concentrations of various bacterial strains. The search was conducted using the keywords “bacteria” and “muramic acid”. We collected data on MurA concentrations from a total of 358 bacterial strains, comprising 208 Gram-positive (GP) bacteria and 150 Gram-negative (GN) bacteria, from 57 articles. The concentrations of these strains were expressed in terms of cell dry weight. For bacterial strains for which only MurA concentrations were reported in cell walls, we converted these values to cell dry weight using a conversion factor of 0.465. Individual data points falling outside 1.5 times the interquartile range were identified as outliers and removed from the box plots. After removing ten outliers for GP bacteria and eight outliers for GN bacteria, the mean value was calculated.

The second dataset is composed of the glucosamine (GlcN) concentrations in fungal strains. The search was conducted using the keywords “fungi” and “glucosamine or hexosamine”. We collected MurA concentration data from a total of 700 bacterial strains from 123 articles. The concentrations of these strains were expressed in terms of cell dry weight. For fungal strains for which only GlcN concentrations in cell walls were reported, we converted these values to cell dry weight using a conversion factor of 0.2 [1]. Individual data points falling outside 1.5 times the interquartile range were identified as outliers and removed from the box plots. After removing ten outliers, the mean value was calculated.

The third dataset included soil MurA and GlcN concentration data. This dataset was compiled from six previous meta-analyses focusing on soil MurA and GlcN concentrations. To avoid duplication, the same articles from different meta-analyses were included only once. In total, we gathered 1604 and 1636 observations of MurA and GlcN concentrations in soils, respectively. Data on ecosystem type, latitude, longitude, mean annual air temperature (MAT), mean annual precipitation (MAP), mean annual potential evaporation (PET), elevation, SOC, soil total nitrogen (TN), soil total phosphorus (TP), microbial biomass C and N (MBC, MBN), pH, and soil texture (clay, silt, and sand) were also collected.

The fourth dataset is composed of data on the ratio of Gram-positive (GP) to Gram-negative (GN) bacteria in soil bacterial communities from various locations worldwide, including our own experiments. The search was conducted using the keywords "bacteria", "ratio", and "Gram". To minimize publication bias, the data were screened based on specific criteria. First, the bacterial biomass was measured using a phospholipid fatty acid approach, and bacterial group identification was performed according to the methods of Joergensen [2]. Second, the GP bacteria were composed of Firmicutes and Actinobacteria based on their phospholipid fatty acids [2]. Third, laboratory-incubated soils were excluded unless a blank treatment was used. Fourth, the latitude and longitude of the soil collection sites were reported in published articles. Fifth, pot experiments were not considered. Sixth, only undisturbed soils were included, and sieved soil was excluded. Seventh, plant litter layers were not included in this dataset. In addition, our own data from cropland soils, comprising 414 observations, were incorporated into this dataset. Overall, a total of 3075 observations of GP:GN ratios from different global locations were included in this dataset. The data were extracted from the graphs using the software GetData (v.2.22).

The fifth dataset we collected is a recent version of the global meta-analysis complied by Guillaume Patoine et al [3]. This dataset is composed of 762 observations of biomass C concentrations of living microbes in soils. It described geographic coordinates, land cover descriptions, sampling data, and other additional environmental variables.

Additional, for some observations in the third, fourth, and fifth datasets, the environmental variables were not reported in the original documents. To address this issue, we filled the missing data according to the sampling year, longitude, and latitude. If the sampling year was not reported from the papers, the five previous years before the publication year was used to extract the environmental values and average them [3]. The missing data were filled in using the following global datasets:

- SOC, TN, TP, pH, and soil texture were obtained from the gridded Global Soil Dataset at a 0.083° spatial resolution. (http://globalchange.bnu.edu.cn/research/soilw).
- MBC and MBN were obtained from the Oak Ridge National Laboratory Distributed Active Archive Center (https://daac.ornl.gov/cgi-bin/dsviewer.pl?ds_id=1264).
- MAT, MAP, and PET were obtained from the monthly CHELSA time series (https://doi.org/10.5061/dryad.kd1d4) . The mean annual value was calculated by the value for each month, with an equal contribution from each month.
- Land cover type layers were obtained from the ESA CCI dataset (https://cds.climate.copernicus.eu) .
- Normalized difference vegetation index (NDVI) were obtained from NOAA’s National Centers for Environmental Information (https://doi.org/10.7289/V5ZG6QH9). Since the data availability coverage of daily NDVI was heterogeneous, a monthly average was taken first, from which an annual average was calculated, with an equal contribution from each month [3].

**Estimation of MDC concentration and stock.** Amino sugar analysis have emerged as the most widespread approach to estimate the MDC concentrations. The central assumption of this approach is that all microbial-derived components are retained in soils to the same extent and the microbial living biomass and necromass have consistent components [1]. Therefore, we can convert from a single component (amino sugars) to MDC at a fixed ratio (i.e., a conversion factor). The conversion factor is calculated by dividing the carbon content of bacterial biomass by the mean amino sugar concentration in microbial strains, assuming that both the bacterial and fungal biomass have approximately 460 mg C g^-1^ dry cell weight [1].

For bacteria, GP bacteria have much more peptidoglycan in the cell wall than GN bacteria [1,4]. Thus, the mean bacterial MurA concentration needs to be weighted by the concentration and weight of the bacteria. Our results showed that the mean MurA concentrations of the GP and GN bacterial strains were 24.1 mg g^-1^ (95% CI: 21.9 to 26.3) and 3.3 mg g^-1^ (95% CI: 3.0 to 3.7), respectively (Fig. S13). Thus, the BDC concentration is estimated as follows:

$$BDC concentration = MurA \times\frac{460}{24.1\times\frac{GP (\%)}{100}+3.3\times\frac{GN (\%)}{100}}$$

where MurA is the concentration of MurA in soils (in mg g^-1^) and GP and GN are the percentages of GP and GN bacteria, respectively, in the soil bacterial community (in percentages).

For fungi, the method employed to determine the FDC concentration followed a similar strategy to that used for bacteria. Our results showed that the mean GlcN concentration in fungal strains was 42.7 mg g^-1^ (95% CI: 40.3 to 45.2). Since soil GlcN is not solely derived from fungi [4,5], the conventional method for estimating fungal-derived GlcN concentrations involves subtracting the bacterial GlcN concentration from the total GlcN concentration in soil, assuming a molar ratio of 1:2 for MurA to GlcN in bacterial cells [4-6]. Thus, the FDC concentration is estimated as follows:

$$FDC concentration= \left( \frac{GlcN}{179.17}-2\times\frac{MurA}{251.23} \right)\times179.17\times\frac{460}{42.7}$$

where GlcN and MurA are the concentrations of GlcN and MurA in the soil (in mg g^-1^), respectively, and 179.17 and 251.23 are the relative molecular masses of GlcN and MurA, respectively.

In addition, the carbon stock can be estimated as follows [3]:

$$C stock=\frac{C concentration}{1000}\times SLT\times BD\times\frac{100-sand (\%)}{100}$$

where C stock is the carbon stock (unit in kg m^-2^), C concentration is the carbon concentration (unit in g kg^-1^), SLT is the soil layer thickness (unit in m), BD is the bulk density (unit in kg m^-3^), and sand is the volumetric fraction of sand in soils (unit in percent).

**Modeling MDC stock dynamics.** To construct a spatial-temporal model of MDC stocks, we calculated the spatial-temporal distributions of MurA and GlcN concentrations and the GP:GN ratio via grid analysis (mentioned above). These spatial-temporal distributions were obtained by the spatial-temporal distributions of climate, vegetation, ecosystems, soils, and microbes. The climate (MAT, MAP, and PET), vegetation (NDVI), and ecosystem (transformed from land use types) data are available for 1981-2018 (mentioned above). Thus, spatial-temporal models of the other variables (SOC, TN, TP, pH, and MBC) need to be constructed first.

We employed ensemble machine learning approaches to construct these spatial-temporal models. Ensemble machine learning can be used to predict a series of interrelated dependent variables. The model sorts the submodels (dependent variables to be predicted) in a specific order, and the results of the last submodel impact the next submodel. This sequential approach improves the learning curve and goodness of fit of subsequent predicted submodels. In our study, the submodels were trained in the order of TP, TN, pH, SOC, and MBC. The reasons for this order are as follows. First, MBC is the sole microbial variable and is usually significantly affected by soil variables [3]. Second, the SOC concentration was used to calculate the contribution of the MDC. Therefore, a submodel that predicts the SOC with a high level of goodness of fit is necessary. Third, pH has been reported to significantly affect the soil GP:GN ratio. Finally, for soil nutrients, predicting TP first provided a better fit to the whole ensemble model than predicting TN first. Overall, we used ensemble machine learning approaches to construct spatial-temporal submodels for four soil variables and one microbial variable. Thirteen spatial-temporal models and 4 spatial models were used to predict the spatial-temporal distributions of MurA and GlcN concentrations and the soil GP:GN ratio (Table S6).

Since all the submodels were constructed using a consistent methodology, we only illustrated the process of constructing the submodel of the TP as an example. We compiled a training set from the third, fourth, and fifth databases. The set included all field-observed TPs but excluded filled TPs from the global environmental layer. The set used TP as the label (dependent variable), three soil textures (sand, clay, and silt), two geographic variables (latitude and elevation), three climatic variables (MAT, MAP, and PET), a vegetation variable (NDVI), and an ecological ecosystem type (note: it is a categorical variable) as the features (independent variable). For continuous variables, we used kernel density estimation to quantify their effects. For the categorical variables, we used confusion matrices to illustrate the correlations between ecosystem type and other variables. The results of the feature importance analysis are shown in Fig. S1d.

Then, we used four conventional machine learning models (the extreme gradient boosting model (XGBoost), the light gradient boosting machine model (LightGBM), the category gradient boosting decision tree model (CatBoost), and the random forest model) and two neural networks [convolutional neural network (CNN) and multilayer perceptron (MLP)] to predict the global distribution of TP and determine the most suitable model. These models have been reported to have good performances in tabular regression tasks. We encoded the "ecosystem type" with one-hot encoding, which transforms each category value into a 0-1 vector, thereby enhancing the model's predictive performance. We then separated the data into features and labels as stated above. We further randomly split the subdataset into a training set and a test set (70%:30%). We employed Optuna, a Python library for hyperparameter optimization. Its optimization algorithm is based on Bayesian optimization. The core of the Bayesian optimization algorithm is to model the objective function using a Gaussian process regression model. This can estimate the expected value and variance of the loss function for each hyperparameter configuration. This is a model-based optimization algorithm that aims to minimize an objective function by learning an agent model. According to this model, the algorithm selects the next hyperparameter configuration to minimize the Bayesian optimal solution of the expected value of the loss function at each iteration. One significant advantage of Bayesian optimization is its ability to automatically determine where to explore the hyperparameter space and where to prune, as well as to efficiently find the optimal solution while avoiding the inefficiencies of random and grid searches. In addition, the agent model for Bayesian optimization can utilize all the historical information about the objective function, thus learning and exploiting prior knowledge about the hyperparameter space during the optimization process. In brief, Optuna offers a simple and effective method for conducting hyperparameter optimization through Bayesian optimization algorithms. Optuna allows us to define the search space for hyperparameters, the objective function, and any necessary constraints, as well as specify an evaluation metric, such as minimizing a loss function. The Bayesian optimization algorithm is used to explore the hyperparameter space, and the best hyperparameter configuration is provided to minimize the objective function. The algorithmic model was tuned using Optuna to minimize the objective function as the mean squared error (MSE) of a fivefold cross-validation. The optimal model is determined by choosing the model with the highest R^2^ and the lowest MSE. Our results showed that the most suitable model for predicting the spatial-temporal distribution of the TP content was the CatBoost machine learning model (Fig. S1a). Based on this optimal model, we predicted the spatial-temporal distribution of the TP content from 1981 to 2018 using gridded data of all the features as a prediction set.

We constructed the next submodel (TN) using the same method. Notably, the TP is a new feature that can be added to the prediction set of TNs. The features of each submodel are listed in Table S6. Figures S1-S8 show the results of each submodel. Based on the results of the submodels, we used the abovementioned formula to calculate the spatial-temporal distributions of the BDC and FDC concentrations. All machine learning was conducted using Python. All mapping was performed using ArcGIS.

It is important to note that there remain some limitations and uncertainties in the data and statistical models. Firstly, we employed a chained ensemble machine learning model to predict a series of variables, considering the interconnections between these variables and significantly enhancing the learning curve and goodness of fit of subsequent predicted submodels. However, this approach may lead to the accumulation of errors from earlier submodels in later predictions, an uncertainty not quantifiable by goodness-of-fit. A networked ensemble machine learning model could mitigate this uncertainty by reducing error transmission and aligning the model structure with soil science knowledge, although it might underperform in later submodels compared to chained models. Therefore, future studies should judiciously select ensemble machine learning model structures based on data availability and model efficacy. Secondly, the majority of data points are concentrated in East Asia, Europe, and North America, which might overrepresent some regions and correspondingly underrepresent others. Although we mark areas of low confidence using representativeness analysis, future collection strategies should ensure balanced data point representation across regions, matching collection efforts with completeness coverage estimates [7,8]. As more studies shed light on global soil MDC distribution, future predictions should incorporate additional research focusing on data-scarce regions.

**Global representativeness assessments.** To determine the spatial extent to which predictions can be made with high confidence, we map each observation in our collected dataset (training set) and each grid location in the global environmental layer (prediction set) into a multi-dimensional space. Specifically, each observation or grid location depends on the value of its corresponding features and is represented as a point in the multi-dimensional space with each feature corresponding to a dimension. For example, in the dataset of amino sugars, we collected a total of 1791 observations of amino sugars and 14 relevant features in situ (latitude, elevation, MAT, MAP, PET, NDVI, SOC, TN, TP, pH, MBC, sand, silt, and clay). We then consider each observation as a vector in a 14-dimensional space. Using this approach, we obtained a total of 1791 vectors from the dataset of amino sugars (from the training set), 3075 vectors from the database of microbial group composition (from the training set), and 44556 vectors from the global environmental layer that needed to be predicted (from the prediction set).

We used two methods to assess the representativeness of our data and model. The dataset of microbial group composition was used as an example for the training set. The first approach uses the Mahalanobis distance between each vector in the prediction set and the center of vectors in the training set (i.e., the vector consisting of the mean of each dimension) after normalization. When a vector in the prediction set has a smaller Mahalanobis distance from the center of vectors, it is closer to the prediction center, and its prediction result has a higher confidence. We set the distance at chisq = 0.975 as the outlier threshold of the Mahalanobis distance [3,7,9]. If the Mahalanobis distance between a vector in the prediction set and the center of vectors is greater than the threshold, this vector is considered an outlier. The second approach defines the area of applicability of the predictions by comparing the dissimilarity index (DI) between the vectors in the prediction set and the vectors in the training set [3,10]. The DI is based on the distance to the nearest neighbor of the vector in multi-dimensional space and is then weighted by the feature importance in the machine learning models. If a vector in the prediction set has a smaller DI value, it is similar to some vectors in the training set and thus can be predicted well. We set the maximum DI of the training data after removing outliers as the threshold for DI. A vector with a DI higher than the threshold is considered an outlier that cannot be predicted with high confidence. As the two approaches function under different principles, they complement each other well and are therefore combined to define the spatial region where model predictions can be applied with confidence.

If a vector in the prediction set passes both the tests of the Mahalanobis distance and the area of applicability, it is close to the prediction center and has similar data in the training set. Thus, the area represented by this vector is defined as the high confidence area. If a vector passes only one test, it is either close to the prediction center or has similar vectors in the training set. It can be predicted very well in theory, but the stability of its prediction results may not be sufficient. Thus, the area represented by this vector is defined as the medium confidence area. If a vector does not pass any test, the area represented by this vector is defined as the low confidence area.

In this study, we set the Antarctic continent and Greenland as unpredictable areas due to the lack of global environmental layer data. Our results showed that the high-, medium-, and low-confidence areas for predicting amino sugar concentrations accounted for 73.1%, 22.2%, and 4.7%, respectively, of the global predictable areas. The high-, medium-, and low-confidence regions for predicting microbial group composition accounted for 90.6%, 5.5%, and 3.9%, respectively, of the global predictable areas.

The estimation of MDC concentrations depends on the amino sugar concentrations and microbial group composition. As a result, when an area is defined as the high confidence area for predicting both the amino sugar concentrations and the microbial composition, the area can be defined as the high confidence area for predicting MDC concentrations. When an area is defined as the medium confidence area or above in both predictions, the area can be defined as the medium confidence area for predicting MDC concentrations. The other areas are defined as low-confidence areas. Our results showed that the high-, medium-, and low-confidence regions for predicting MDC concentrations accounted for 72.6%, 20.8%, 6.6%, and 3.9%, respectively, of the global predictable areas (Fig. S11).

Notably, the quantity of data points significantly influences the results of the area of applicability test but has minimal impact on the Mahalanobis distance outcomes. Some regions with scarce or no data, such as in North America, Eurasia, the southern tips of South America, and Africa, possess mild climates and common soil conditions. Thus, these regions are likely proximate to the prediction center and share similar climatic and soil conditions with the data points in our dataset. These regions easily pass the two tests and are classified as high confidence areas. Conversely, regions like the Tibetan Plateau often exhibit unique climatic or soil conditions, positioning them distant from the prediction center. As a result, these regions struggle to pass the Mahalanobis distance test but readily pass the area of applicability test due to the relatively large number of data points. Hence, these regions are typically categorized as medium confidence areas.

**Quantifying the response of the MDC concentration to warming.** Linear mixed-effects (LME) models were used to quantify the response of the MDC concentration to warming in this study. LME models, which are a type of generalized linear model, generally incorporate fixed and random effects. Fixed effects are used to explain overall differences within the sample, while random effects allow for the consideration of individual-level variation. This modeling approach is more realistic and flexible than traditional linear models and makes better use of data in many practical applications. In contrast, traditional linear models include only fixed effects and errors and cannot account for the influence of random effects. Traditional linear models deviate from actual situations in scenarios where individual differences need to be considered. Therefore, the LME model has been widely used to quantify the response of C dynamics to temperature change [11-13].

It is well known that MAT changes over time and space. Considering only the period from 1981 to 2018, the change in MAT due to spatial changes was much greater than that due to temporal changes. As shown in Figs. S9 and S10, high latitudes with low MATs generally have higher MDC concentrations in soils. This negative correlation may confound the effect of temporal-induced MAT changes on MDC concentrations. Therefore, we used the MDC concentration as the dependent variable, MAT as the fixed effect, and site as a random effect, nesting the site-level relationship within the overall relationship. After accounting for individual differences at each site, our results showed that MAT still had a significantly negative effect on MDC concentrations on a global scale (Fig. 2). This suggested that increasing temperature due to temporal changes can decrease MDC concentrations on a global scale.

To determine whether it was necessary to include the random effects corresponding to the variations in slope and/or intercept among sites, we assessed the improvement in model fitness between the null model (no random effect) and three alternative models (random effect on the slope only, random effect on the intercept only, and random effect on the slope and intercept). We compared the Akaike information criterion (AIC) values of these models (Table S1) and found that the random effects model, which best described the changes in MDC concentration, included random variations in both the slope and intercept. Therefore, we employed a random-effects structure including random variation in both the slope and intercept to assess the significance of the fixed effects. In addition, the confidence interval overlap method was used to compare the slopes and intercepts generated from different mixed-effects models (for example, comparing the response of BDC and FDC concentrations to warming). All LME model analyses were performed with the ‘lmer’ function in the ‘lme4’ package in R statistical software (v.3.6.3).

**Projections of MDC stocks under different shared socioeconomic pathways.** The shared socioeconomic pathway (SSP) is a scenario framework developed collaboratively by the International Council for Science and the Intergovernmental Panel on Climate Change. Its purpose is to provide a common baseline for climate models by integrating changes in social, economic, and energy systems to assess and compare the impacts of climate change. The SSP divides the trajectory of human societal development in the 21^st^ century into five scenarios representing different social, economic, and energy development tendencies. These scenarios included the sustainability scenario (SSP126), the middle scenario (SSP245), the regional rivalry scenario (SSP370), and the fossil-fueled development scenario (SSP585). The SSP scenario framework enables climate models to more accurately simulate future climate change and provide baseline scenarios for policy-making and climate adaptation planning.

To predict future MDC stocks, we mapped the global distributions of MAT projections for 2040-2100 under the SSP126, SSP245, SSP370, and SSP585 scenarios based on data from the Coupled Model Intercomparison Project (Phase 6) downscaled future climate projections and the IPSL-CM6A-LR climate model from the Inter-Sectoral Impact Model Intercomparison Project [14]. We assumed that the effect of the MDC stock on temperature change in the future century would be consistent with that in previous decades. We then projected the global distribution of MDC stock predictions for 2040-2100 under the four SSP scenarios based on the MDC stock predictions in 2018.

**References for Materials and Methods**

1. Appuhn A and Joergensen RG. Microbial colonisation of roots as a function of plant species. *Soil Biol Biochem* 2006; **38**: 1040-1051.

2. Joergensen RG. Phospholipid fatty acids in soil-drawbacks and future prospects. *Biol Fertil Soils* 2022; **58**: 1-6.

3. Patoine G, Eisenhauer N, Cesarz S *et al*. Drivers and trends of global soil microbial carbon over two decades. *Nat Commun* 2022; **13**: 4195.

4. Whalen ED, Grandy AS, Sokol NW *et al*. Clarifying the evidence for microbial- and plant-derived soil organic matter, and the path toward a more quantitative understanding. *Glob Chang Biol* 2022; **28**: 7167-7185.

5. Engelking B, Flessa H and Joergensen RG. Shifts in amino sugar and ergosterol contents after addition of sucrose and cellulose to soil. *Soil Biol Biochem* 2007; **39**: 2111-2118.

6. Liang C, Amelung W, Lehmann J *et al*. Quantitative assessment of microbial necromass contribution to soil organic matter. *Glob Chang Biol* 2019; **25**: 3578-3590.

7. Guerra CA, Heintz-Buschart A, Sikorski J *et al*. Blind spots in global soil biodiversity and ecosystem function research. *Nat Commun* 2020; **11**: 3870.

8. Chao A and Jost L. Coverage-based rarefaction and extrapolation: standardizing samples by completeness rather than size. *Ecology* 2012; **93**: 2533-2547.

9. Guerra CA, Delgado-Baquerizo M, Duarte E *et al*. Global projections of the soil microbiome in the Anthropocene. *Glob Ecol Biogeogr* 2021; **30**: 987-999.

10. Meyer H and Pebesma E. Predicting into unknown space? Estimating the area of applicability of spatial prediction models. *Methods Ecol Evol* 2021; **12**: 1620-1633.

11. Chen HY, Xu X, Fang CM *et al*. Differences in the temperature dependence of wetland CO_2_ and CH_4_ emissions vary with water table depth. *Nat Clim Change* 2021; **11**: 766-771.

12. Hu H, Chen J, Zhou F *et al*. Relative increases in CH_4_ and CO_2_ emissions from wetlands under global warming dependent on soil carbon substrates. *Nat Geosci* 2024; **17**: 26-31.

13. Yvon-Durocher G, Allen AP, Bastviken D *et al*. Methane fluxes show consistent temperature dependence across microbial to ecosystem scales. *Nature* 2014; **507**: 488-491.

14. Warszawski L, Frieler K, Huber V *et al*. The Inter-Sectoral Impact Model Intercomparison Project (ISI-MIP): Project framework. *Proc Natl Acad Sci USA* 2014; **111**: 3228-3232.


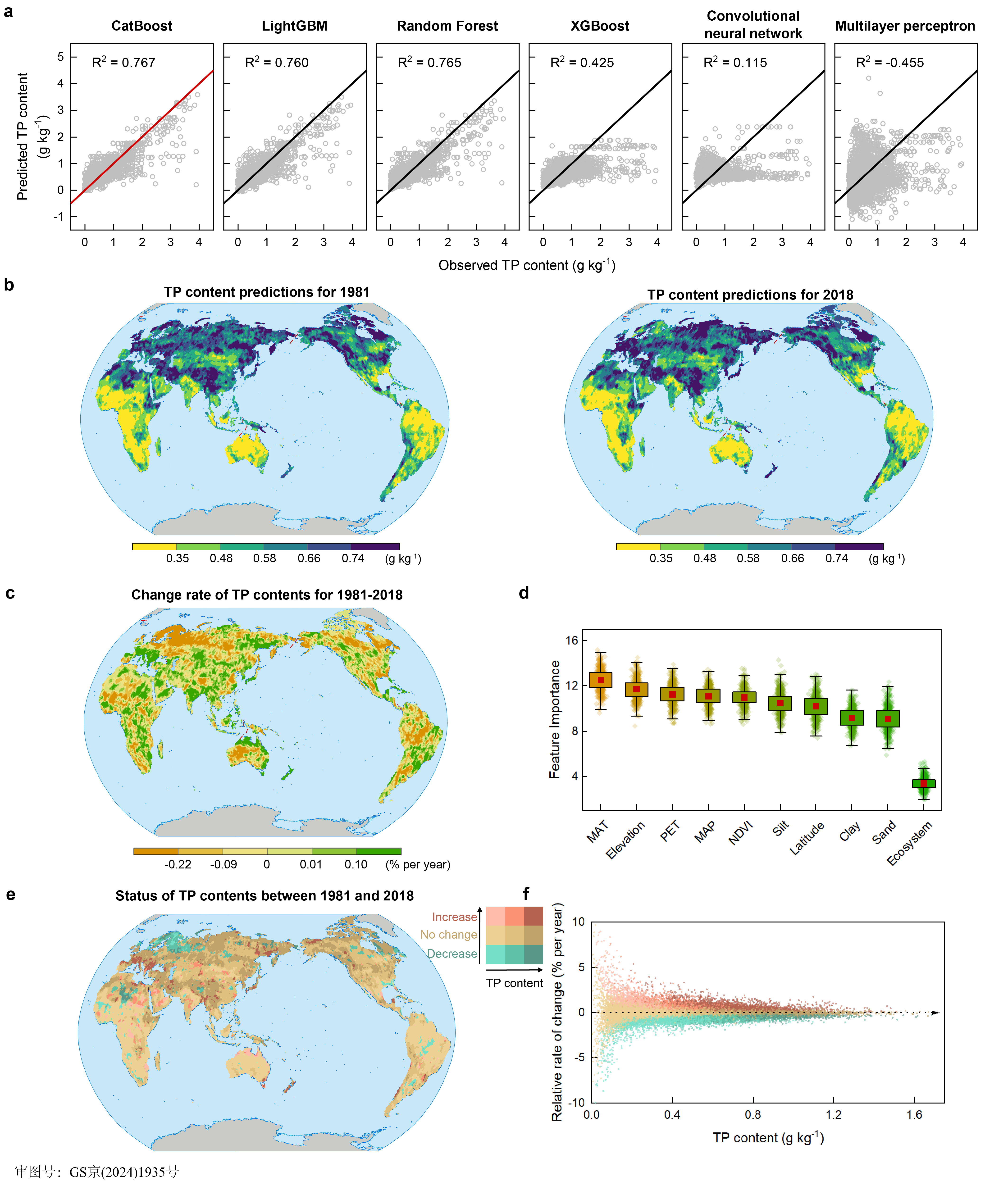


**Supplementary Fig. 1. Predicted spatial distributions and temporal trends of the soil total phosphorus (TP) content. a,** Comparison of the prediction results of different machine learning models. The red lines indicate the 1:1 line. R^2^ represents the coefficient of determination, and the optimal model is characterized by the maximum R^2^. **b,** Global maps of the predicted TP content for 1981 and 2018. **c,** Relative percentage change in TP content per year. **d,** Feature importance. The importance from 100 models runs with different random seeds, calculated by the mean decrease in accuracy after variable permutation. **e,** Status of the TP content between 1981 and 2018. Bivariate plot comparing the relative TP content rate of change (% per year) against the quantity of the TP content. The status categories for the rate of change were determined using confidence intervals, while the TP content status groups were established based on quantile distributions (divided into three equal parts). **f,** Distribution and classification of point values from the locations in panel e.


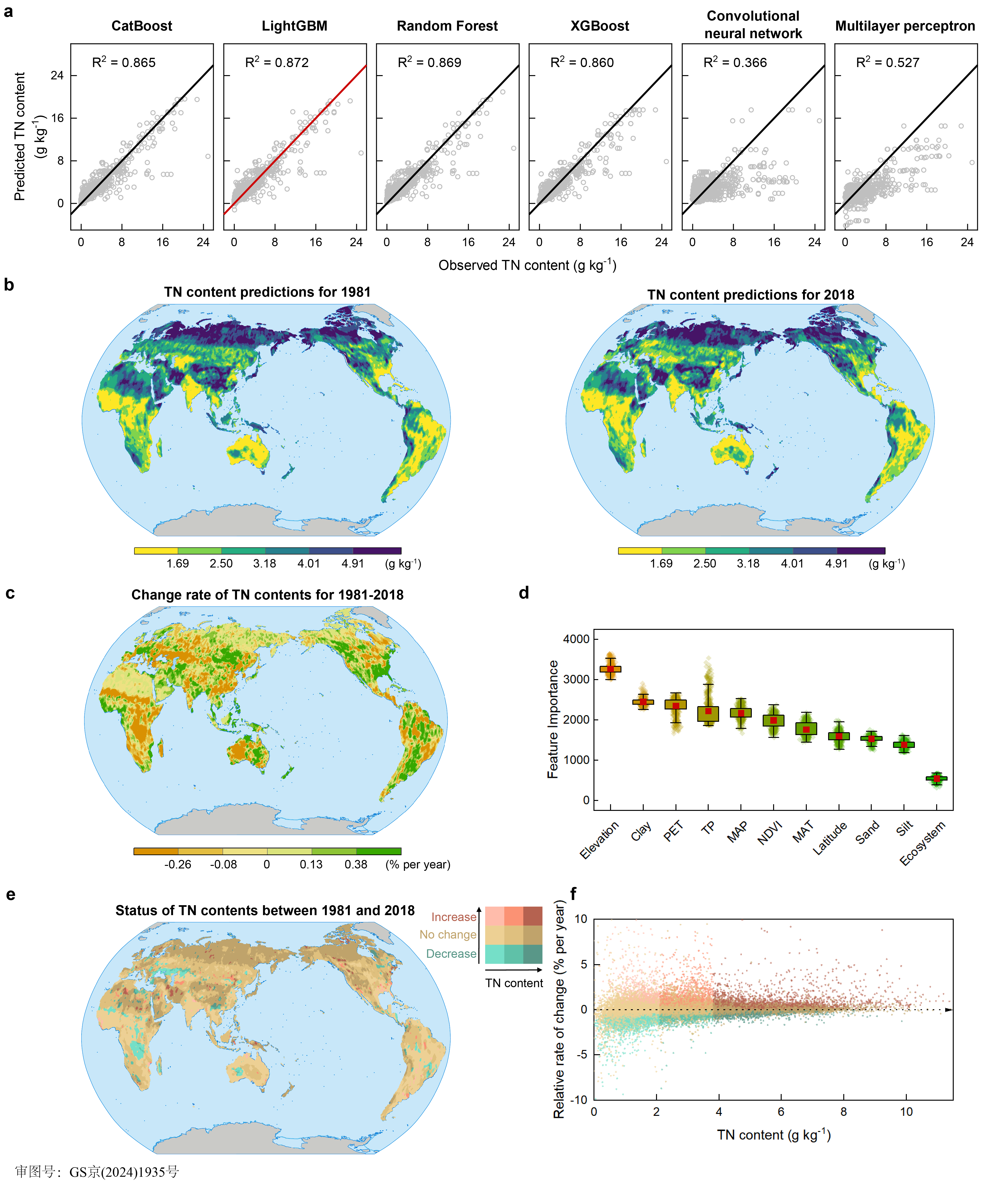


**Supplementary Fig. 2. Predicted spatial distributions and temporal trends of the soil total nitrogen (TN) content. a,** Comparison of the prediction results of different machine learning models. The red lines indicate the 1:1 line. R^2^ represents the coefficient of determination, and the optimal model is characterized by the maximum R^2^. **b,** Global maps of the predicted TN content for 1981 and 2018. **c,** Relative percentage change in TN content per year. **d,** Feature importance. The importance from 100 models runs with different random seeds, calculated by the mean decrease in accuracy after variable permutation. **e,** Status of the TN content between 1981 and 2018. Bivariate plot comparing the relative TN content rate of change (% per year) against the quantity of TN content. The status categories for the rate of change were determined using confidence intervals, while the TN content status groups were established based on quantile distributions (divided into three equal parts). **f,** Distribution and classification of point values from the locations in panel e.


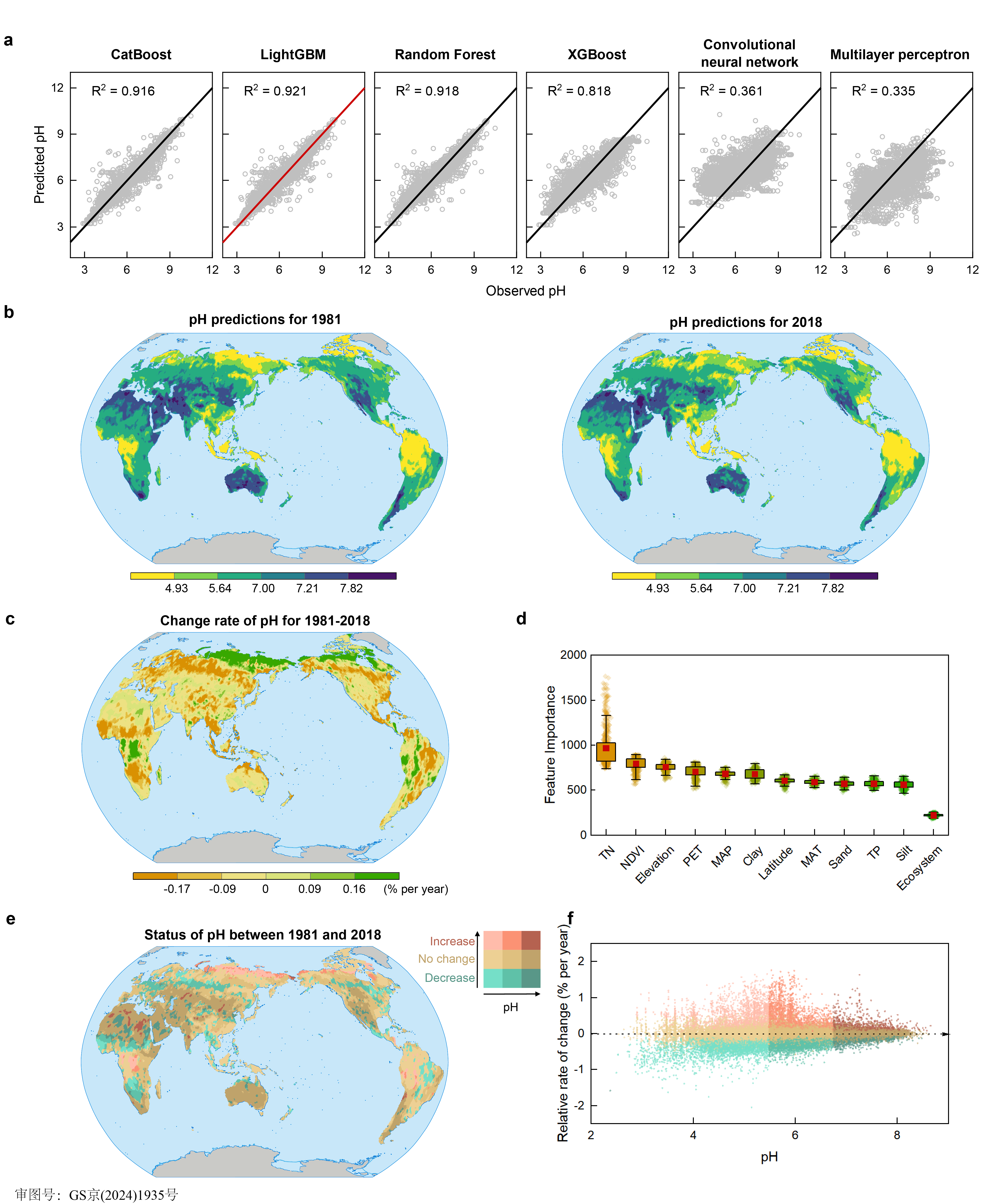
**Supplementary Fig. 3. Predicted spatial distributions and temporal trends of soil pH. a,** Comparison of the prediction results of different machine learning models. The red lines indicate the 1:1 line. R^2^ represents the coefficient of determination, and the optimal model is characterized by the maximum R^2^. **b,** Global maps of the predicted pH for 1981 and 2018. **c,** Relative percentage change in pH per year. **d,** Feature importance. The importance from 100 models runs with different random seeds, calculated by the mean decrease in accuracy after variable permutation. **e,** Status of the pH between 1981 and 2018. Bivariate plot comparing the relative pH rate of change (% per year) against the value of pH. The status categories for the rate of change were determined using confidence intervals, while the pH status groups were established based on quantile distributions (divided into three equal parts). **f,** Distribution and classification of point values from the locations in panel e.


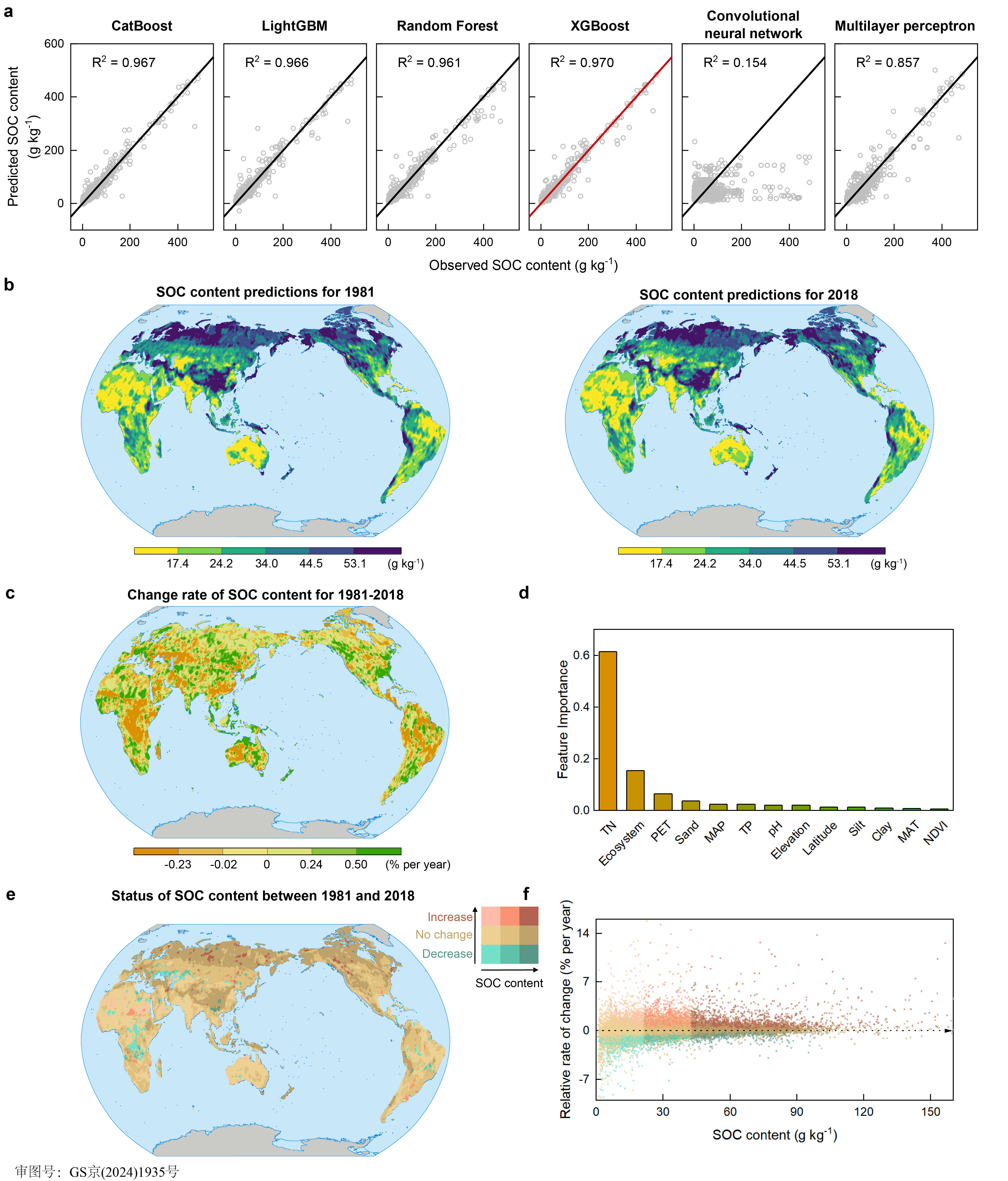


**Supplementary Fig. 4. Predicted spatial distributions and temporal trends of soil organic carbon (SOC) content. a,** Comparison of the prediction results of different machine learning models. The red lines indicate the 1:1 line. R^2^ represents the coefficient of determination, and the optimal model is characterized by the maximum R^2^. **b,** Global maps of the predicted SOC content for 1981 and 2018. **c,** Relative percentage change in SOC content per year. **d,** Feature importance. Due to the lack of random seeds in the XG Boost model, the feature importance is unique. **e,** Status of the SOC content between 1981 and 2018. Bivariate plot comparing the relative SOC content rate of change (% per year) against the quantity of SOC. The status categories for the rate of change were determined using confidence intervals, while the SOC status groups were established based on quantile distributions (divided into three equal parts). **f,** Distribution and classification of point values from the locations in panel e.


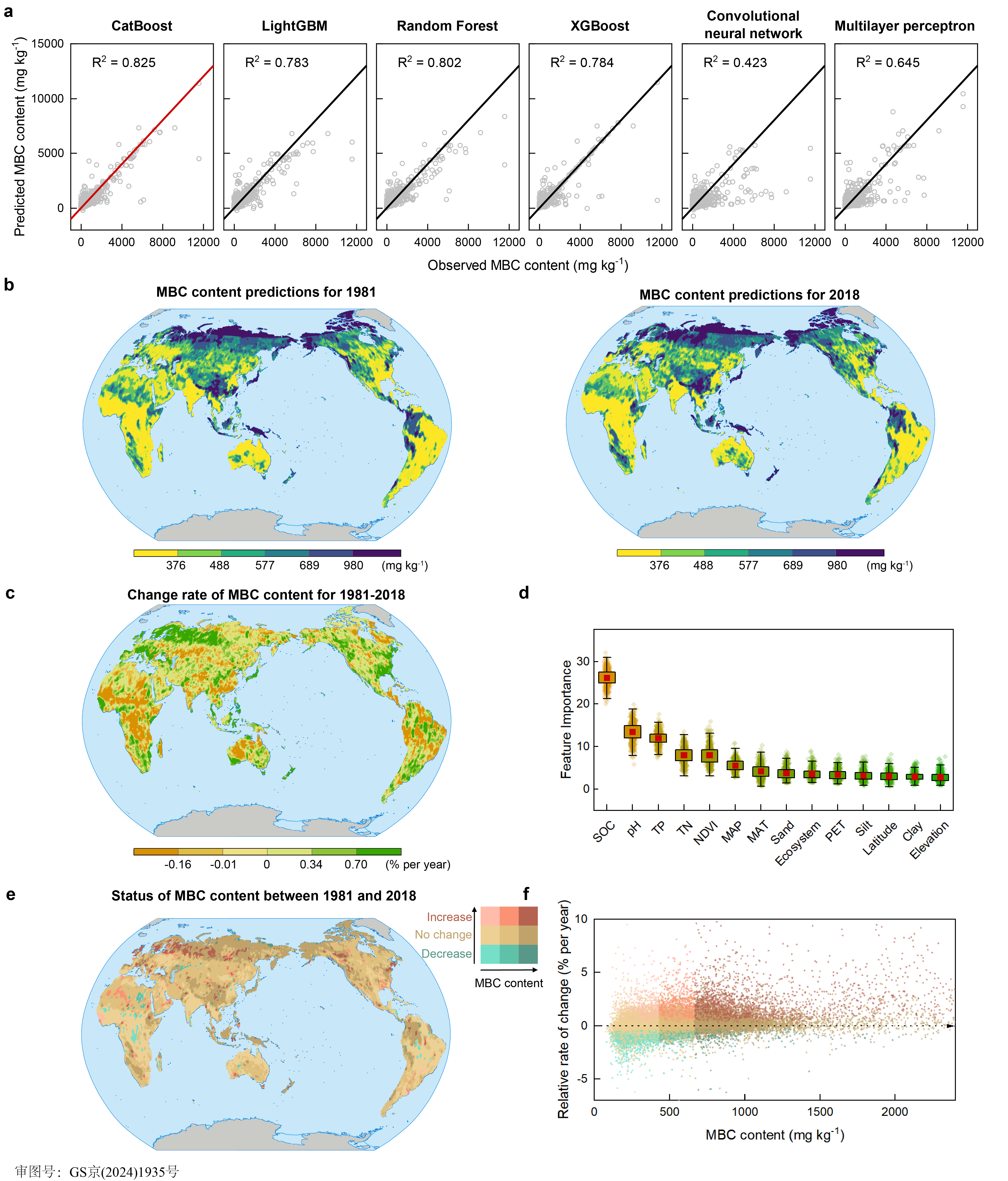


**Supplementary Fig. 5. Predicted spatial distributions and temporal trends of the soil microbial biomass carbon (MBC) content. a,** Comparison of the prediction results of different machine learning models. The red lines indicate the 1:1 line. R^2^ represents the coefficient of determination, and the optimal model is characterized by the maximum R^2^. **b,** Global maps of the predicted MBC content for 1981 and 2018. **c,** Relative percentage change in the MBC content per year. **d,** Feature importance. The importance from 100 models runs with different random seeds, calculated by the mean decrease in accuracy after variable permutation. **e,** Status of the MBC content between 1981 and 2018. Bivariate plot comparing the relative MBC content percentage change (% per year) against the quantity of MBC. The status categories for the rate of change were determined using confidence intervals, while the MBC status groups were established based on quantile distributions (divided into three equal parts). **f,** Distribution and classification of point values from the locations in panel e.


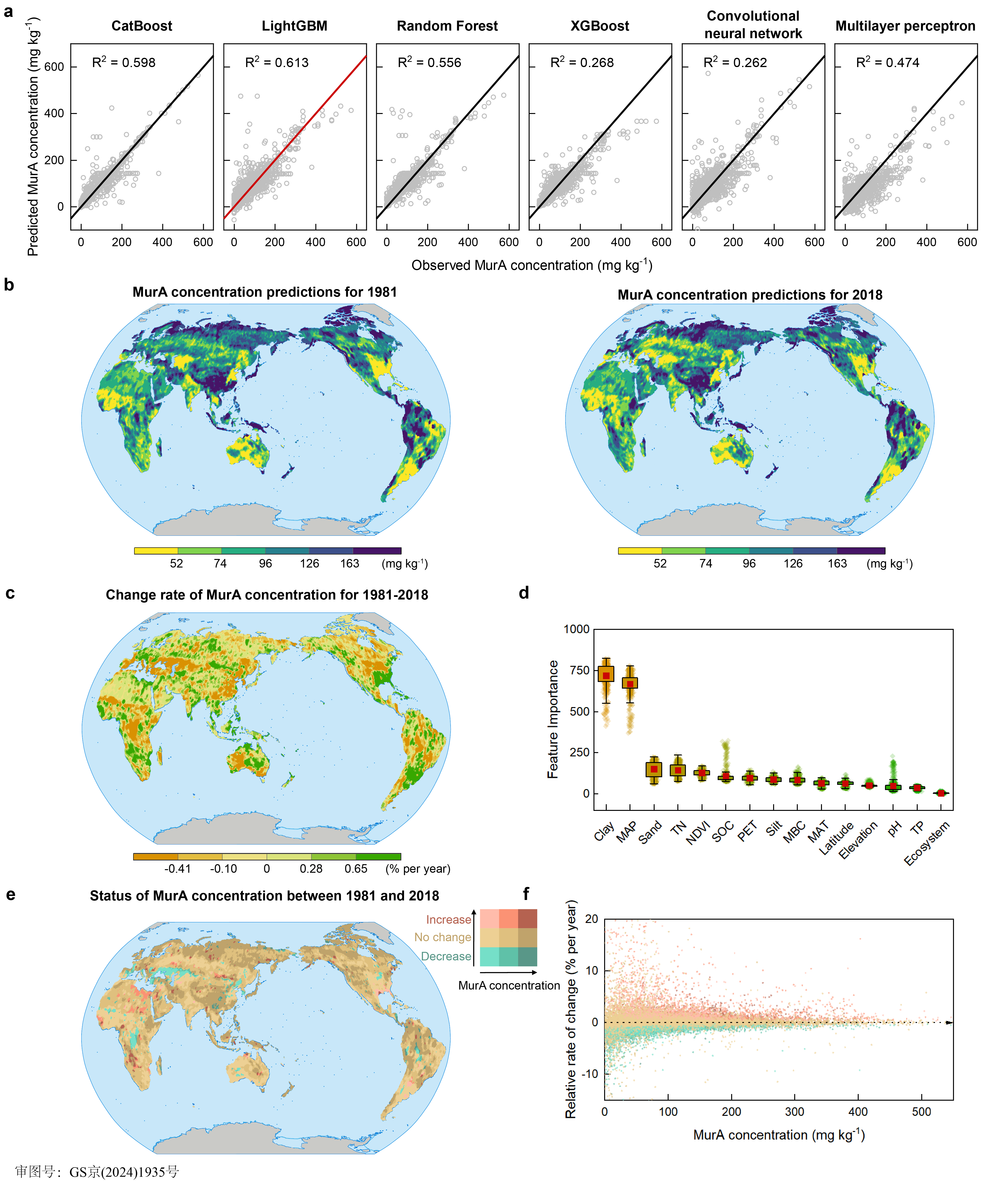


**Supplementary Fig. 6. Predicted spatial distributions and temporal trends of soil muramic acid (MurA) concentration. a,** Comparison of the prediction results of different machine learning models. The red lines indicate the 1:1 line. R^2^ represents the coefficient of determination, and the optimal model is characterized by the maximum R^2^. **b,** Global maps of the predicted MurA concentration for 1981 and 2018. **c,** Relative percentage change in MurA concentration per year. **d,** Feature importance. The importance from 100 models runs with different random seeds, calculated by the mean decrease in accuracy after variable permutation. **e,** Status of the MurA concentration between 1981 and 2018. Bivariate plot comparing the relative MurA concentration rate of change (% per year) against the quantity of MurA. The status categories for the rate of change were determined using confidence intervals, while the MurA concentration status groups were established based on quantile distributions (divided into three equal parts). **f,** Distribution and classification of point values from the locations in panel e.


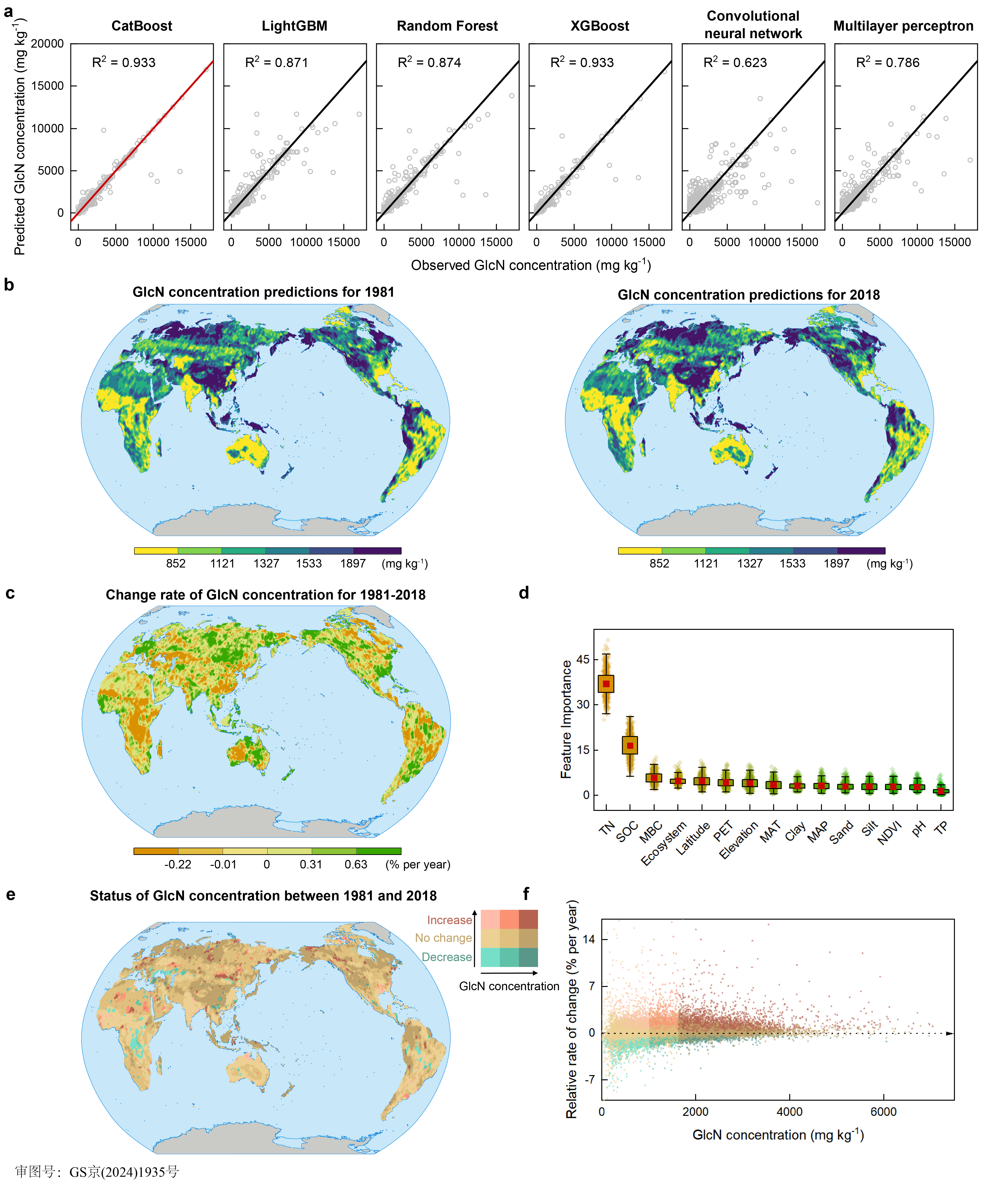


**Supplementary Fig. 7. Predicted spatial distributions and temporal trends of soil glucosamine (GlcN) concentration. a,** Comparison of the prediction results of different machine learning models. The red lines indicate the 1:1 line. R^2^ represents the coefficient of determination, and the optimal model is characterized by the maximum R^2^. **b,** Global maps of the predicted GlcN concentration for 1981 and 2018. **c,** Relative percentage change in GlcN concentration per year. **d,** Feature importance. The importance from 100 models runs with different random seeds, calculated by the mean decrease in accuracy after variable permutation. **e,** Status of the GlcN concentration between 1981 and 2018. Bivariate plot comparing the relative GlcN concentration rate of change (% per year) against the quantity of GlcN. The status categories for the rate of change were determined using confidence intervals, while the GlcN concentration status groups were established based on quantile distributions (divided into three equal parts). **f,** Distribution and classification of point values from the locations in panel e.


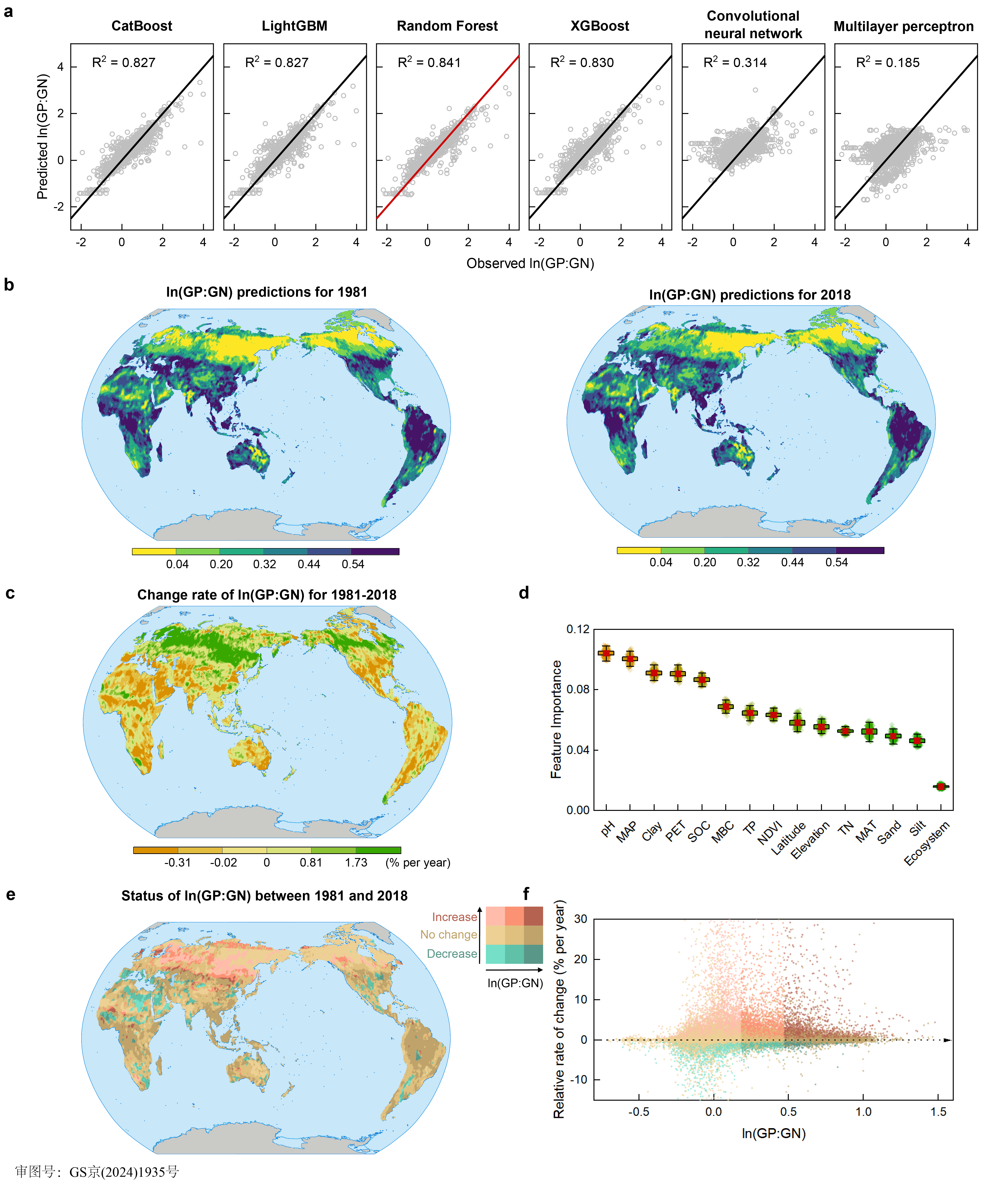


**Supplementary Fig. 8. Predicted spatial distributions and temporal trends of the natural logarithm of the ratio of gram-positive to gram-negative bacteria [ln(GP:GN)] in soil bacterial communities. a,** Comparison of the prediction results of different machine learning models. The red lines indicate the 1:1 line. R^2^ represents the coefficient of determination, and the optimal model is characterized by the maximum R^2^. **b,** Global maps of the predicted ln(GP:GN) for 1981 and 2018. **c,** Relative percentage change in ln(GP:GN) per year. **d,** Feature importance. The importance from 100 models runs with different random seeds, calculated by the mean decrease in accuracy after variable permutation. **e,** Status of ln(GP:GN) between 1981 and 2018. Bivariate plot comparing the relative ln(GP:GN) rate of change (% per year) against the value of ln(GP:GN). The status categories for the rate of change were determined using confidence intervals, while the ln(GP:GN) status groups were established based on quantile distributions (divided into three equal parts). **f,** Distribution and classification of point values from the locations in panel e.


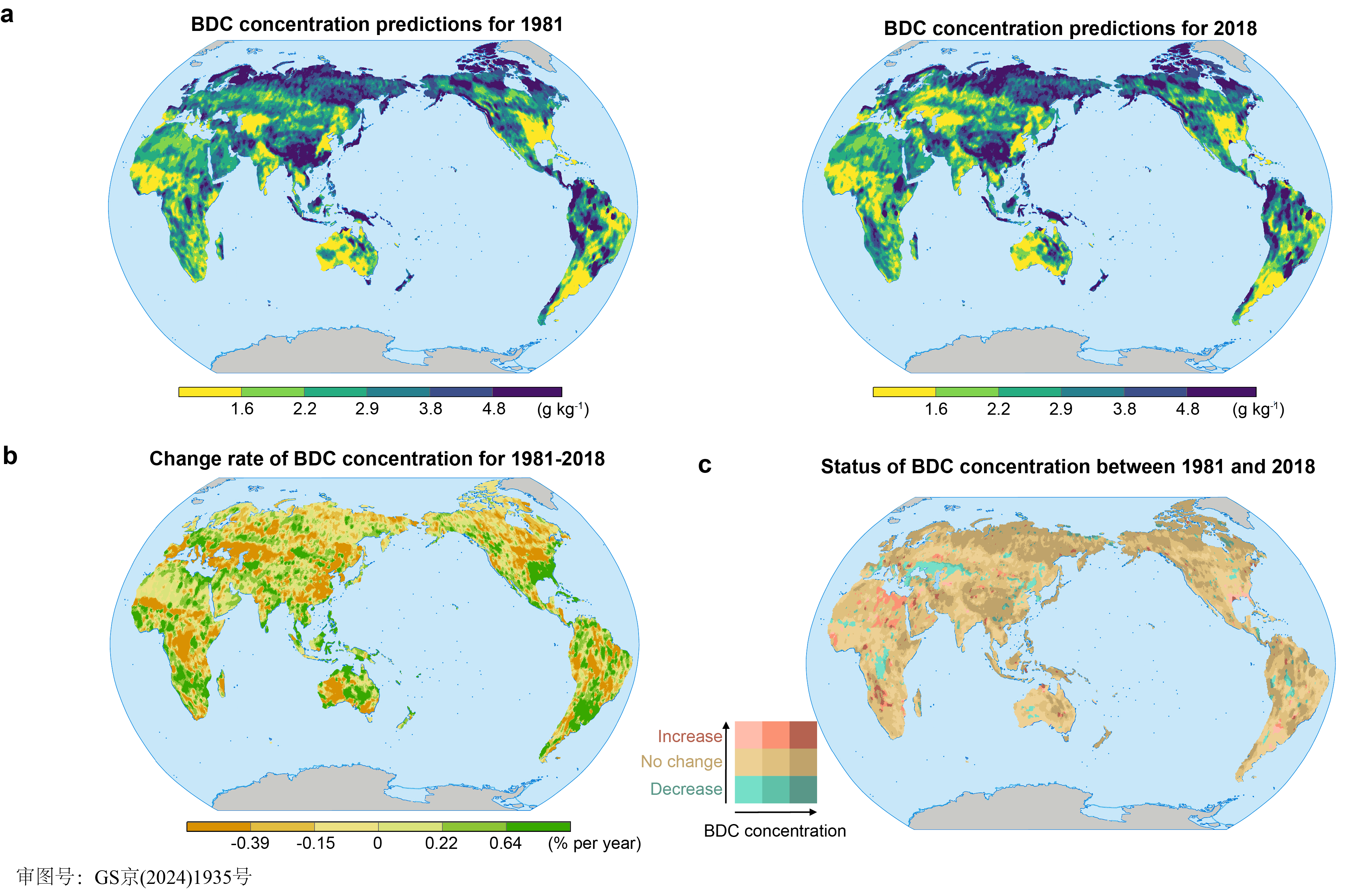


**Supplementary Fig. 9. Predicted spatial distributions and temporal trends of bacterial-derived carbon (BDC) concentration. a,** Global map of the predicted BDC concentrations for 1981 and 2018. **b,** Relative rates of change in the BDC concentration as percentages per annum. **c,** Status of the BDC concentration between 1981 and 2018. Bivariate plot comparing the relative rate of change in BDC concentration (% per year) against the quantity of BDC concentration. The status categories for the rate of change were determined using confidence intervals, while the BDC concentration status groups were established based on quantile distributions (divided into three equal parts).


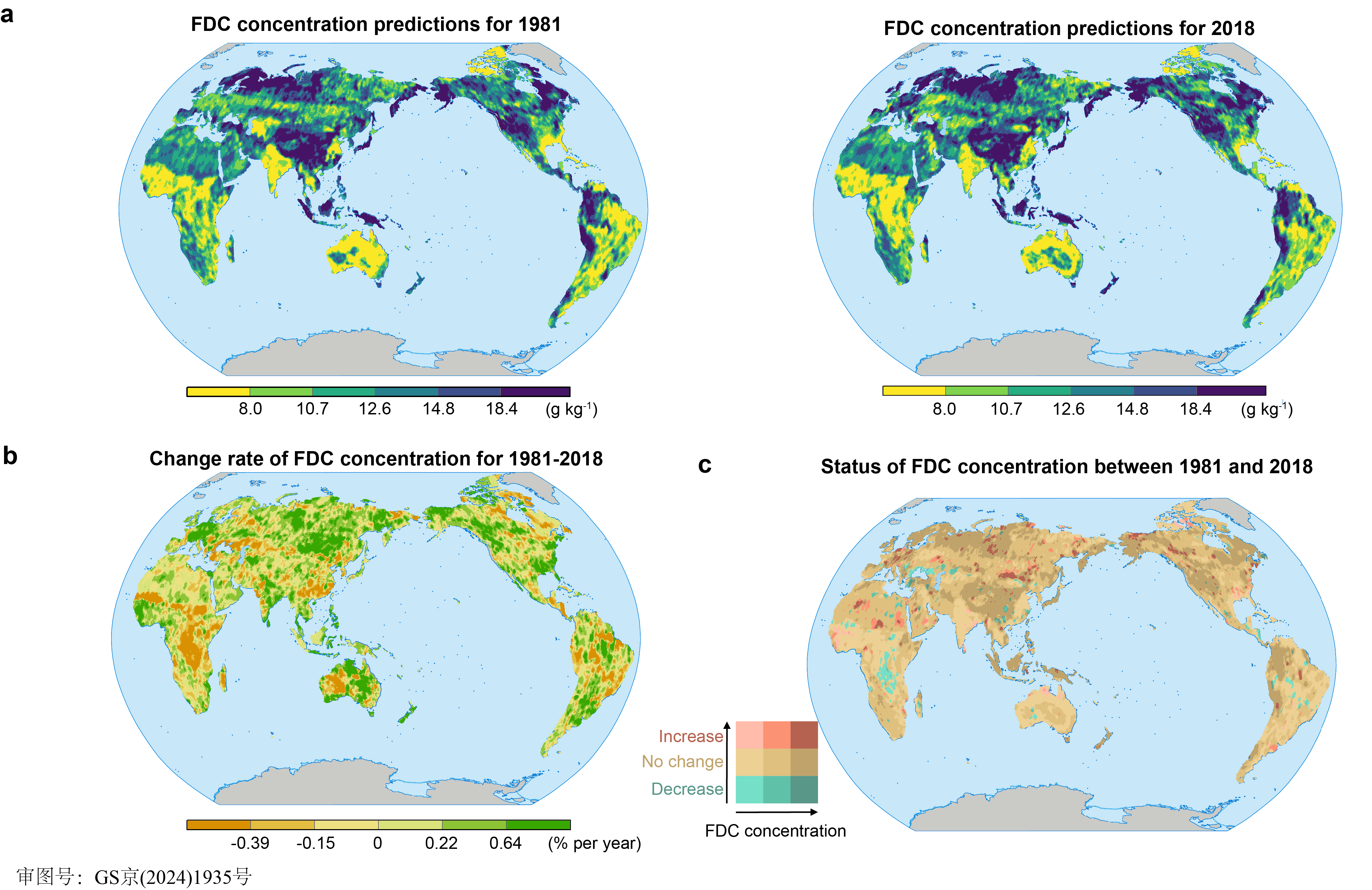


**Supplementary Fig. 10. Predicted spatial distributions and temporal trends of fungal-derived carbon (FDC) concentration. a,** Global map of the predicted FDC concentration for 1981 and 2018. **b,** Relative rates of change in FDC concentration as percentages per annum. **c,** Status of the FDC concentration between 1981 and 2018. Bivariate plot comparing the relative rate of change in FDC concentration (% per year) against the quantity of FDC concentration. The status categories for the rate of change were determined using confidence intervals, while the FDC concentration status groups were established based on quantile distributions (divided into three equal parts).


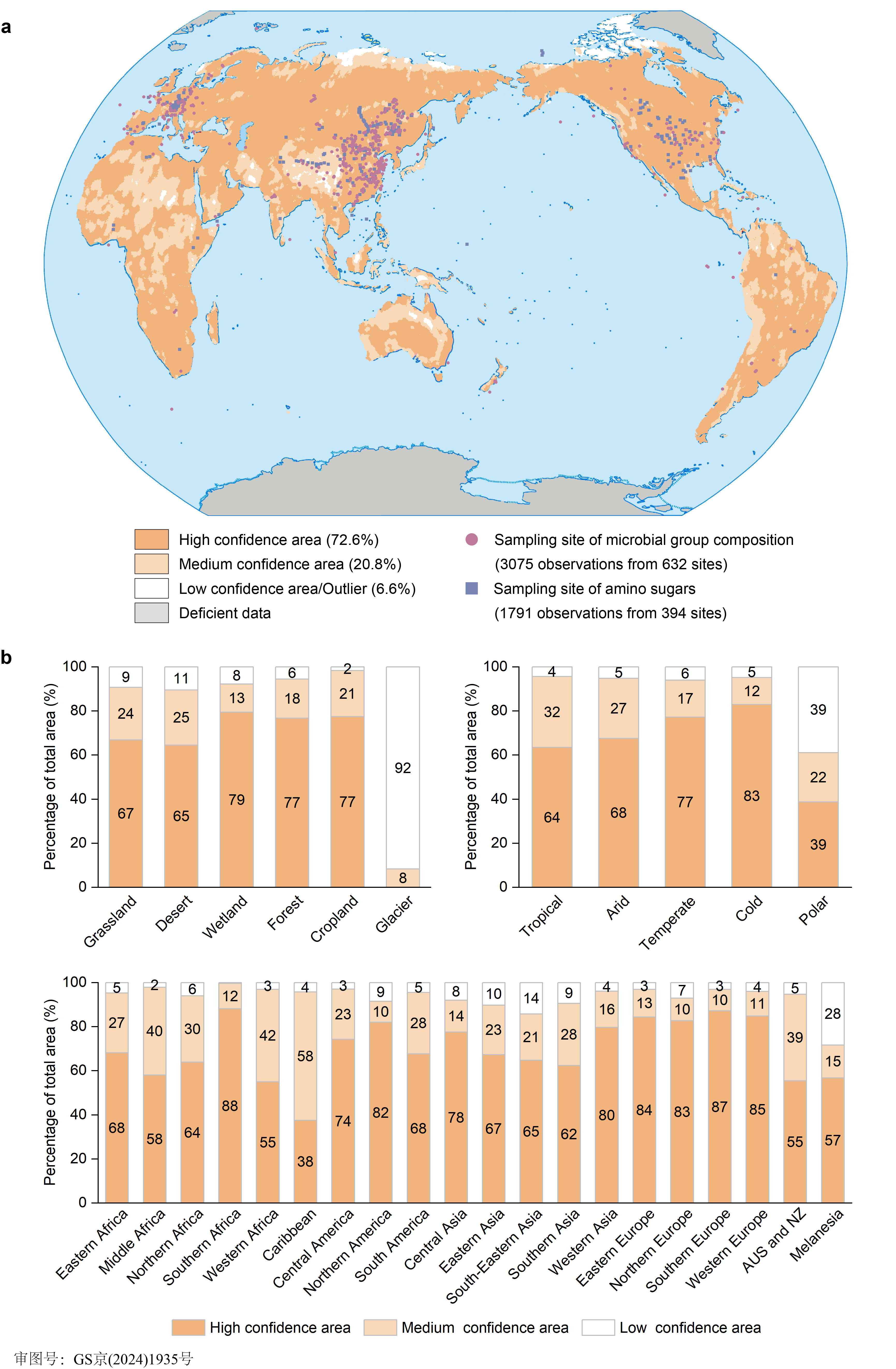


**Supplementary Fig. 11. The areas with high, medium, and low confidence for predicting MDC concentrations.** a, The areas with different confidence levels for predictions are described by different colors. The numbers in brackets indicate the percentage of the global predictable area. The red and blue points are the microbial group composition (3075 observations from 632 sites) and amino sugar (1791 observations from 394 sites) sampling sites, respectively. b, The percentages of high-, medium-, and low-confidence areas among the global predictive areas for different ecosystem types, climatic zones, and regions. The climatic zones are classified by the Köppen climate classification.


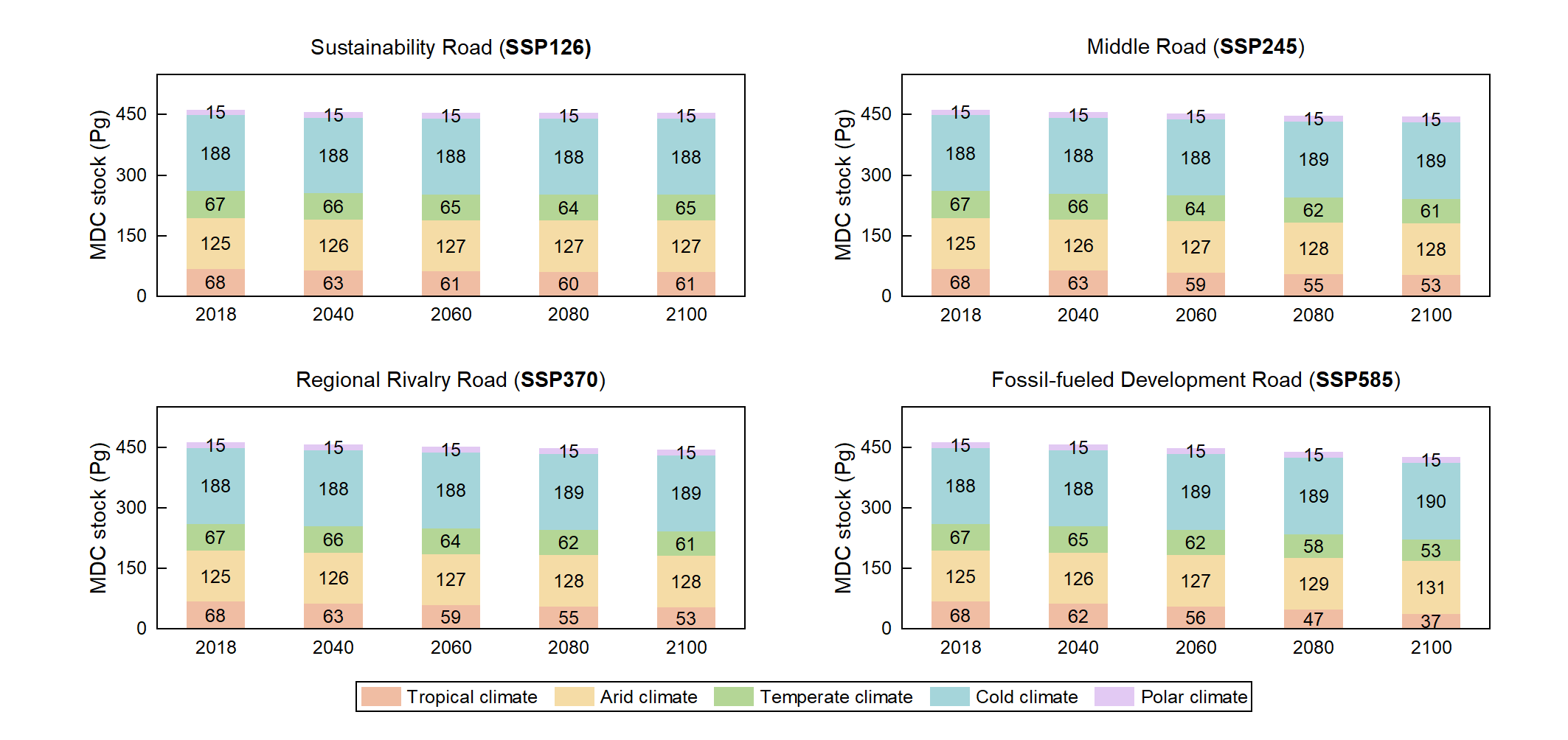


**Supplementary Fig. 12. Predictions of microbial-derived carbon (MDC) stocks under different shared socioeconomic pathways.** The classification of climates was based on the Köppen climate classification.


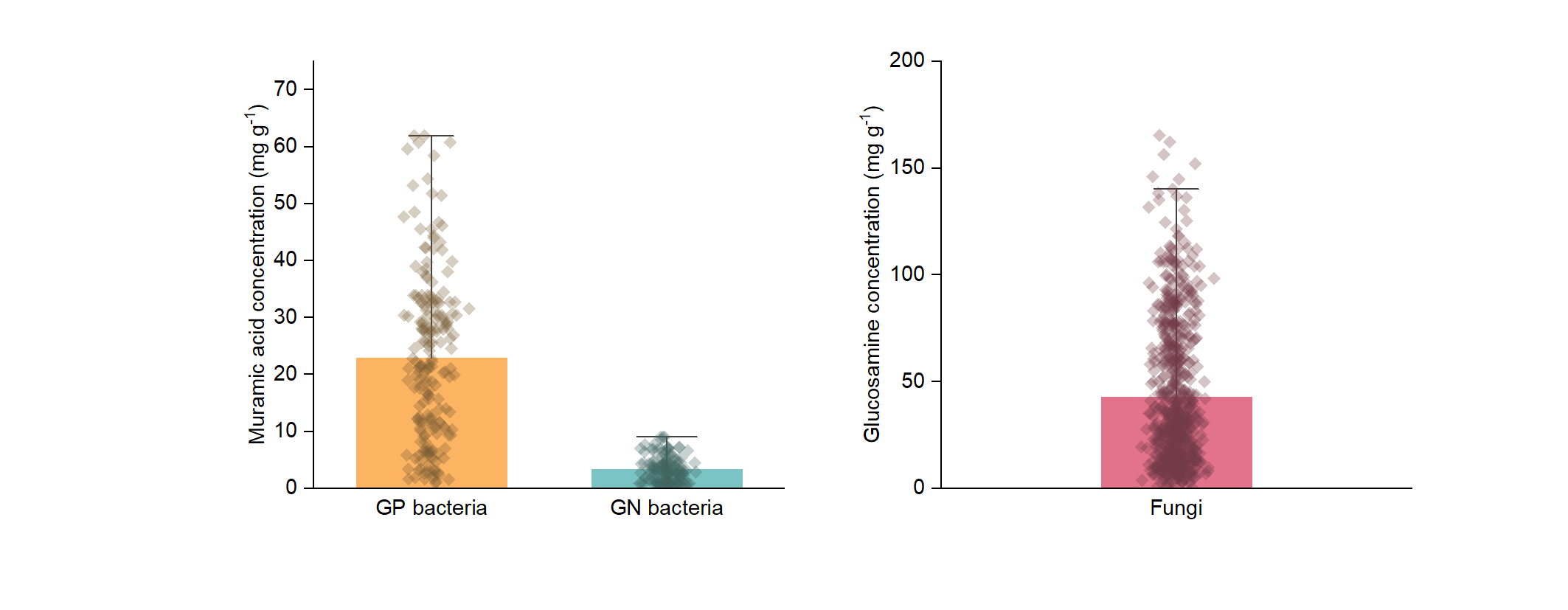


**Supplementary Fig. 13. Muramic acid concentrations in bacterial strains and glucosamine concentrations in fungal strains.** GP and GN bacteria refer to Gram-positive and Gram-negative bacteria, respectively. The data are represented as the mean and 1.5 times the interquartile ranges.

**Supplementary Table 1. Comparison among linear mixed effect models with different random effects.**

| Dependent variable | Random effect | | | |
| --- | --- | --- | --- | --- |
|  | No effect | On slope  only | On intercept  only | On slope and intercept |
| MDC | 10634709 | 9083161 | 9332947 | **9080391** |
| MDC/SOC | 14941351 | 14130946 | 14142506 | **14114140** |
| SOC | 13007326 | 11238923 | 11557283 | **11234760** |
| BDC | 7592042 | 5906940 | 6002136 | **5901802** |
| FDC | 10114203 | 8633906 | 8886395 | **8630213** |
| FDC/BDC | 24123528 | 24115910 | 24101496 | **24101073** |

The numbers in this table indicate the AIC (information-theoretic Akaike information criterion). The model with the minimum AIC value is the optimal model. In these models, the mean annual air temperature is the fixed effect, and site is the random effect. The bold text indicates the model with the minimal AIC. MDC, microbial-derived carbon; BDC, bacterial-derived carbon; FDC, fungal-derived carbon; SOC, soil organic carbon.

**Supplementary Table 2. Warming effects on microbial-derived carbon concentrations in different climates.**

|  | Slope  (g kg^-1^ per °C) | 95% confidence interval | *p* | n | Site |
| --- | --- | --- | --- | --- | --- |
| Global | -0.180 | -0.185 to -0.174 | <0.001 | 1539792 | 41616 |
| Tropic | -1.019 | -1.146 to -1.039 | <0.001 | 255596 | 6908 |
| Arid | 0.111 | 0.090 to 0.133 | <0.001 | 445369 | 12037 |
| Temperate | -0.565 | -0.626 to -0.504 | <0.001 | 211048 | 5704 |
| Cold | 0.025 | 0.011 to 0.040 | <0.001 | 580049 | 15677 |
| Polar | 0.001 | 0.001 to 0.002 | <0.001 | 47730 | 1290 |

The slope values and 95% confidence intervals were calculated by linear mixed-effects models. The random effects for all the models in this table are on the slope and intercept.

**Supplementary Table 3. Warming effects on fungal-derived carbon concentrations in different climatic and ecosystem types.**

|  | Slope  (g kg^-1^ per °C) | 95% confidence interval | *p* | n | Site |
| --- | --- | --- | --- | --- | --- |
| Global | -0.140 | -0.144 to -0.135 | <0.001 | 1539792 | 41616 |
| Tropic | -0.746 | -0.787 to -0.705 | <0.001 | 255596 | 6908 |
| Arid | 0.053 | 0.035 to 0.071 | <0.001 | 445369 | 12037 |
| Temperate | -0.345 | -0.388 to -0.303 | <0.001 | 211048 | 5704 |
| Cold | 0.095 | 0.082 to 0.107 | <0.001 | 580049 | 15677 |
| Polar | 0.083 | 0.049 to 0.116 | <0.001 | 47730 | 1290 |

The slope values and 95% confidence intervals were calculated by linear mixed-effects models. The random effects for all the models in this table are on the slope and intercept.

**Supplementary Table 4. Warming effects on the bacterial-derived carbon concentration in different climatic and ecosystem types.**

|  | Slope  (g kg^-1^ per °C) | 95% confidence interval | *p* | n | Site |
| --- | --- | --- | --- | --- | --- |
| Global | -0.059 | -0.060 to -0.057 | <0.001 | 1539792 | 41616 |
| Tropic | -0.337 | -0.369 to -0.307 | <0.001 | 255596 | 6908 |
| Arid | 0.054 | 0.049 to 0.058 | <0.001 | 445369 | 12037 |
| Temperate | -0.150 | -0.175 to -0.138 | <0.001 | 211048 | 5704 |
| Cold | -0.061 | -0.065 to -0.058 | <0.001 | 580049 | 15677 |
| Polar | -0.094 | -0.101 to -0.087 | <0.001 | 47730 | 1290 |

The slope values and 95% confidence intervals were calculated by linear mixed-effects models. The random effects for all the models in this table are on the slope and intercept.

**Supplementary Table 5. Warming effects on the concentration ratio of fungal to bacterial-derived carbon in different climatic and ecosystem types.**

|  | Slope  (g kg^-1^ per °C) | 95% confidence interval | *p* | n | Site |
| --- | --- | --- | --- | --- | --- |
| Global | *NA* | *NA* | 0.269 | 1539792 | 41616 |
| Tropic | *NA* | *NA* | 0.926 | 255596 | 6908 |
| Arid | *NA* | *NA* | 0.766 | 445369 | 12037 |
| Temperate | *NA* | *NA* | 0.765 | 211048 | 5704 |
| Cold | 0.145 | 0.054 to 0.235 | 0.002 | 580049 | 15677 |
| Polar | 0.102 | 0.091 to 0.112 | <0.001 | 47730 | 1290 |

The slope values and 95% confidence intervals were calculated by linear mixed-effects models. The random effects for all the models in this table are on the slope only. NA indicates a nonsignificant model (*p* > 0.05).

**Supplementary Table 6. Features of the training sets for each model in ensemble machine learning.**

| Dependent variable | Feature | | | | | | | | | | | | | | |
| --- | --- | --- | --- | --- | --- | --- | --- | --- | --- | --- | --- | --- | --- | --- | --- |
|  | Climate | | | | | Geography | | | | | Soil | | | | |
|  | MAT | MAP | PET | NDVI | Eco. | Lat. | Ele. | Sand | Silt | Clay | TP | TN | pH | SOC | MBC |
| TP | **√** | **√** | **√** | **√** | **√** | **√** | **√** | **√** | **√** | **√** |  |  |  |  |  |
| TN | **√** | **√** | **√** | **√** | **√** | **√** | **√** | **√** | **√** | **√** | **√** |  |  |  |  |
| pH | **√** | **√** | **√** | **√** | **√** | **√** | **√** | **√** | **√** | **√** | **√** | **√** |  |  |  |
| SOC | **√** | **√** | **√** | **√** | **√** | **√** | **√** | **√** | **√** | **√** | **√** | **√** | **√** |  |  |
| MBC | **√** | **√** | **√** | **√** | **√** | **√** | **√** | **√** | **√** | **√** | **√** | **√** | **√** | **√** |  |
| GP:GN | **√** | **√** | **√** | **√** | **√** | **√** | **√** | **√** | **√** | **√** | **√** | **√** | **√** | **√** | **√** |
| MurA | **√** | **√** | **√** | **√** | **√** | **√** | **√** | **√** | **√** | **√** | **√** | **√** | **√** | **√** | **√** |
| GlcN | **√** | **√** | **√** | **√** | **√** | **√** | **√** | **√** | **√** | **√** | **√** | **√** | **√** | **√** | **√** |

The red checkmark indicates the feature has both temporal and spatial distributions. The black checkmark indicates the feature has only a spatial distribution and its spatial distribution is constant from 1981 to 2018. MAT, mean annual air-temperature; MAP, mean annual precipitation; PET, mean annual potential evaporation; NDVI, normalized difference vegetation index; Eco., ecosystem type; Lat., latitude; Ele., elevation; SOC, soil organic carbon; TN, soil total nitrogen; TP, soil total phosphorus; MBC, living microbial biomass carbon.
